# Regioselective Insertion of Aluminum(I) in the *cyclo*‐P_5_ Ring of Pentaphosphaferrocene

**DOI:** 10.1002/anie.202002774

**Published:** 2020-04-19

**Authors:** Ravi Yadav, Thomas Simler, Bhupendra Goswami, Christoph Schoo, Ralf Köppe, Subhayan Dey, Peter W. Roesky

**Affiliations:** ^1^ Institute of Inorganic Chemistry Karlsruhe Institute of Technology (KIT) Engesserstrasse 15 76131 Karlsruhe Germany

**Keywords:** aluminum, ferrocene, polyphosphides, regioselectivity, triple-decker

## Abstract

A route to directly access mixed Al–Fe polyphosphide complexes was developed. The reactivity of pentaphosphaferrocene, [Cp*Fe(*η* ^5^‐P_5_)] (Cp*=C_5_Me_5_), with two different low‐valent aluminum compounds was investigated. The steric and electronic environment around the [Al^I^] centre are found to be crucial for the formation of the resulting Al–Fe polyphosphides. Reaction with the sterically demanding [Dipp‐*BDI*Al^I^] (Dipp‐*BDI*={[2,6‐^*i*^Pr_2_C_6_H_3_NCMe]_2_CH}^−^) resulted in the first Al‐based neutral triple‐decker type polyphosphide complex. For [(Cp*Al^I^)_4_], an unprecedented regioselective insertion of three [Cp*Al^III^]^2+^ moieties into two adjacent P−P bonds of the *cyclo*‐P_5_ ring of [Cp*Fe(η^5^‐P_5_)] was observed. The regioselectivity of the insertion reaction could be rationalized by isolating an analogue of the reaction intermediate stabilized by a strong σ‐donor carbene.

## Graphical abstract

**Stepping into the ring**: Low‐valent aluminum reacts with pentaphosphaferrocene allowing direct access to Al–Fe heterometallic polyphosphides (see structure; white C, light blue Al, dark blue Fe, pink P). The ligands around the Al atoms are crucial for the formation of Al–Fe polyphosphides. The experimental and theoretical studies give insight into the reaction pathway.

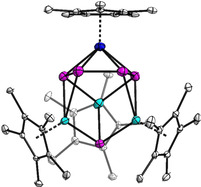

The discovery of ferrocene [Cp_2_Fe] (Cp=η^5^‐C_5_H_5_) in 1951 led to a fundamental change in organometallic chemistry.^1^ The isolobal analogy between Cp^−^ and *cyclo*‐P_5_^−^ steered interest in using *cyclo*‐P_5_^−^ as a ligand for the synthesis of sandwich‐type complexes.^2^ In a seminal report in 1987, Scherer and Brück synthesized pentaphosphaferrocene, [Cp*Fe(η^5^‐P_5_)] (Cp*=C_5_Me_5_), by co‐thermolysis of white phosphorous and [Cp*Fe(CO)_2_]_2_.^3^ Scheer and co‐workers have engaged in using [Cp*Fe(η^5^‐P_5_)] as a tool to access inorganic supramolecules and polymers by taking advantage of the phosphorus lone pairs on the *cyclo*‐P_5_ ring.^4^ Apart from using [Cp*Fe(η^5^‐P_5_)] in inorganic polymer chemistry, understanding the reactivity of [Cp*Fe(η^5^‐P_5_)] towards nucleophiles and different reducing agents has also attracted recent attention.^5^ The redox properties of [Cp*Fe(η^5^‐P_5_)] were studied by cyclic voltammetry^6^ and synthetically.5a, 5b, 5c, 5e

It is interesting to explore the scope of air‐stable starting materials as sources for poly‐pnictogen species as alternatives to the conventionally used and highly reactive P_4_.^7^ Recently, we have shown that the *cyclo*‐P_5_ ring of [Cp*Fe(η^5^‐P_5_)] could be used as a polyphosphorous source. The reaction of [Cp*Fe(η^5^‐P_5_)] with [LSiCl] (L=PhC(N^t^Bu)_2_) resulted in the sila‐phosphaferrocene, [η^4^‐P_4_SiL‐FeCp*], via substitution of one P atom by an isoelectronic [LSi] fragment.^8^

Recently, the organometallic chemistry of mono‐valent aluminum compounds, which was pioneered in the 1990s,^9^ has witnessed renewed interest,^10^ which can be attributed to their ability to activate small molecules and organic substrates featuring single, double, or triple bonds.9l, 11 The reactivity of [Al^I^] is not limited to organic substrates: main‐group elements, such as S_8_,^12^ Se,9b and Te,9b have been used to make aluminum heterocyclic complexes. Monovalent aluminum complexes have also been used to access rare Al–P cages and clusters by reducing white phosphorous.12a, 13 In general, phosphorous containing heterocyclic compounds can be prepared by derivatization of the highly reactive P_4_ cage. We were challenged to examine the reactivity of air stable [Cp*Fe(η^5^‐P_5_)] with [Al^I^] complexes to obtain Al polyphosphorous complexes. The reactivity pattern of [Al^I^] is known to be highly dependent on the type of ligands used to stabilize the monovalent aluminum centre.9l, 11a, 11c, 11d, 14, 15 Therefore, using different electronic and steric environments on [Al^I^] complexes may lead to different types of activation of [Cp*Fe(η^5^‐P_5_)], such as conformational changes or controlled fragmentation of the *cyclo*‐P_5_ ring.

Herein, we report on the reactivity of [Cp*Fe(η^5^‐P_5_)] with two different monovalent aluminum complexes. We have isolated the first examples of Al–Fe‐based neutral triple‐decker polyphosphides. Also, the insertion of three [Cp*Al^III^]^2+^ moieties into P−P bonds led to the isolation of an unprecedented Al–Fe polyphosphide complex containing four metal centres. The possible intermediate for the insertion of [Cp*Al^III^]^2+^ moieties in the *cyclo*‐P_5_ ring was trapped by using a nucleophilic carbene.

The reaction between equimolar amounts of [Cp*Fe(η^5^‐P_5_)] and [Dipp‐*BDI*Al^I^]9j in toluene at room temperature resulted in the formation of [(Dipp‐*BDI*Al^III^)(μ,η^3^:η^4^‐P_5_)FeCp*] (**1**) in 35 % yield (Dipp‐*BDI*={[2,6‐^*i*^Pr_2_C_6_H_3_NCMe]_2_CH}^−^; Scheme 1). During the reaction, the aluminum atom is oxidized to give [Al^III^] while [Cp*Fe(η^5^‐P_5_)] is reduced twice. As a result, the *cyclo*‐P_5_ ring loses its 6 π‐electron aromaticity, resulting in a conformational change from planar to envelope‐shaped.5b, 5e The ^1^H NMR spectrum of **1** showed one single resonance for the Cp* methyl protons, shifted downfield from *δ*=1.08 ppm (in [Cp*Fe(η^5^‐P_5_)]) to *δ*=1.22 ppm. Also, three new broad resonances at *δ*=98.4, 60.7, and 32.1 ppm were observed in the ^31^P{^1^H} NMR spectrum at room temperature, suggesting a fluxional behaviour of the *cyclo*‐P_5_ ring. A well‐resolved ^31^P{^1^H} NMR spectrum could be recorded at −40 °C showing an AA′MXX′ spin system with multiplets at *δ*=97.2 (P_XX′_), 60.6 (P_M_), and 30.7 (P_AA′_) ppm apparent for the formation of an envelope conformation of the *cyclo*‐P_5_ ring (Figure 1 and Table S1 in the Supporting Information). The molecular structure of **1** in the solid state revealed the formation of a triple‐decker type complex with a bent *cyclo*‐P_5_ ring (Figure 2). To our knowledge, **1** is the first example of an Al‐containing neutral triple‐decker heterometallic polyphosphide complex. The Al−P1 (2.3231(15) Å) and Al−P4 (2.465(2) Å) bond lengths are in the reported range of Al−P single bonds (2.308(2) to 2.422(2) Å),12a, 13 whereas the Al–P5 separation (2.784(2) Å) is relatively long indicating only a weak coordination.^16^ The P2−P3 (2.1647(14) Å) and P3−P4 (2.186(2) Å) bond lengths are shorter than the P1−P2 (2.223(2) Å), P1−P5 (2.2145(14) Å) and P4−P5 (2.2735(14) Å) analogues, which is a result of the elongation of P−P bonds upon coordination to aluminum. This is in line with the theoretically calculated shared electron numbers (SEN) given in the Supporting Information. A similar trend has been observed in a samarium polyphosphide complex.5e The reduction of [Cp*Fe(η^5^‐P_5_)] by [Dipp‐*BDI*Al^I^] complex is in sharp contrast with the reported reactivity of [Cp*Fe(η^5^‐P_5_)] with cationic [Ga^I^] and [Tl^I^] species. In the case of [M^I^(Al{OC(CF_3_)_3_}_4_)] (M=Tl and Ga), coordination polymers featuring a planar *cyclo*‐P_5_, [{M^I^(Al{OC(CF_3_)_3_}_4_)} (μ,η^5^:η^5^:η^1^‐P_5_)FeCp*] were obtained.^17^ This anomalous trend in reactivity can be mainly attributed to the higher reductive ability of [Al^I^] complexes as compared to [Ga^I^] and [Tl^I^] analogues.

**Figure 1:**
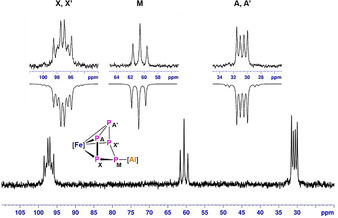
^31^P{^1^H} NMR spectrum (162 MHz, 233 K, [D_8_]toluene) of compound **1** with nuclei assigned to an AA′MXX′ spin system; insets: extended signals (upward) and simulations (downward).

**Figure 2:**
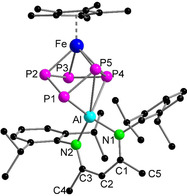
Molecular structure of **1** in the solid state. H atoms are omitted for clarity. CCDC numbers of all the structures reported herein are available in the Supporting Information.

**Scheme 1:**
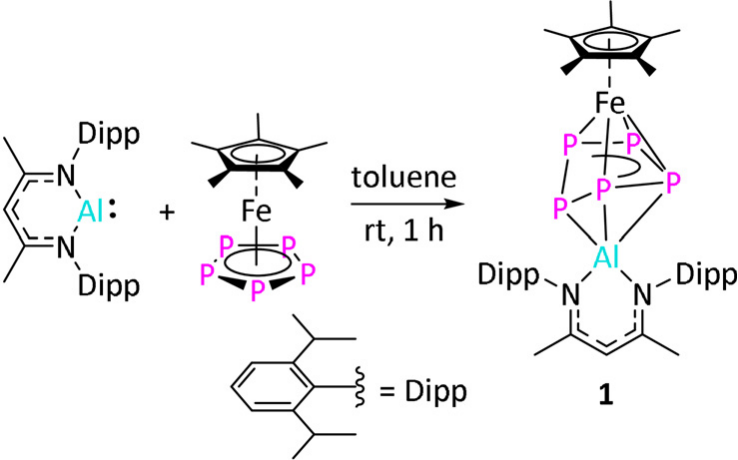
Synthesis of complex **1**.

As the reactivity of monovalent aluminum complexes highly depends on the coordination environment around the aluminum centre (see above), we decided to examine the reactivity of another low‐valent aluminum complex, that is, [(Cp*Al^I^)_4_].9a, 9b The reaction between [(Cp*Al^I^)_4_] and [Cp*Fe(η^5^‐P_5_)] in toluene resulted in the formation of complex [(μ_3_‐P)(Cp*Al^III^)_2_{P_4_(Al^III^Cp*)} (FeCp*)] (**2**) irrespective of the stoichiometric ratio and the reaction conditions. The solid‐state structure of **2** confirmed the formation of an unprecedented Al–Fe polyphosphide complex containing four metal centres (Figure 3). Formally, the *cyclo*‐P_5_‐ring has been six‐fold reduced by three equivalents of [Cp*Al^I^] forming one P_4_^4−^ and one P^3−^ unit, which are charged balanced by three [Cp*Al^III^]^2+^ and one [Cp*Fe]^+^ moiety. However, theoretical calculations show an electron distribution, which is more complex (see below). The [4+1] fragmentation of the *cyclo*‐P_5_ ring is very rare.^18^ The reaction of [LSi‐SiL] with [Cp*Fe(η^5^‐P_5_)] showed a similar fragmentation, however, in this case, a seven‐membered Si‐P ring, [η^4^‐P_5_(SiL)_2_‐FeCp*], was obtained.^8^

**Figure 3:**
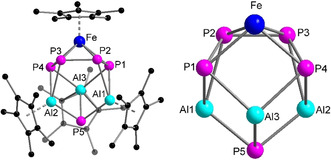
Molecular structure of **2** (left) in the solid state and view of **2** (right) without the Cp* moieties for a clearer view of the core structure.

On the basis of the identity of **2**, the yield of the complex could be increased to 47 % by using the optimised conditions (Scheme 2). The reaction mixture needs to be heated for 7 days at 80 °C to ensure the purity of complex **2**. We have noticed that during prolonged heating all the minor side‐products decompose and precipitate from the toluene solution, hence facilitating the isolation of complex **2** in a pure form. As illustrated in Figure 3, one [Cp*Fe]^+^ unit is η^4^‐coordinated to one *cyclo*‐P_4_(AlCp*) moiety, where the average Fe−P bond length is slightly longer than that in [Cp*Fe(η^5^‐P_5_)] (2.317 vs. 2.273 Å, respectively).5b The *cyclo*‐P_4_(AlCp*) unit is bound in a η^4^‐mode to [Cp*Fe]^+^ as well as η^2^‐coordinated to two [Cp*Al^III^]^2+^ units. In addition, a P atom is bound to the ring. The Cp*‐rings bind in a η^5^‐fashion to Al1 and Al2 whereas Al3 is only η^3^‐coordinated. Al1 and Al2 also bind in η^2^‐mode to the *cyclo*‐P_4_(AlCp*) moiety and are further coordinated to the terminal P5. The average Al−P5 bond length (2.316(2) Å) is in the usual range of Al−P single bonds (2.308(2) to 2.422(2) Å),12a, 16 as are the Al3−P1 and Al3−P4 bonds. However, the Al1−P1 (2.476(2) Å), Al1−P2 (2.676(2) Å), Al2−P3 (2.678(2) Å), and Al2−P4 (2.517(2) Å) bonds are longer than usual Al−P single bonds, which suggests a weaker coordination.12a, 13, 16 In addition, there are weak Al–Al interactions with short Al–Al separations (Al1−Al3 (2.911(2)) and Al2−Al3 (2.919(2) Å).^13^ The average P−P bond length (2.191(2) Å) in the *cyclo*‐P_4_(AlCp*) unit is shorter than a P−P single bond, indicating the presence of a partial double‐bond character.12a, 16

**Scheme 2:**
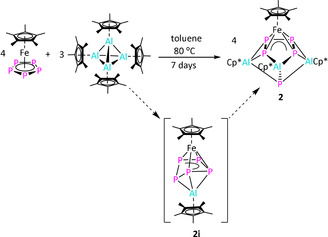
Synthesis of complex **2** via intermediate **2 i**.

In contrast to the non‐equivalent character of the three aluminum centres of **2** in the solid state, only two sharp singlets at *δ*=2.16 ppm (45 H, Cp* on Al atoms) and *δ*=1.30 ppm (15 H, Cp* on Fe atom) were observed in the ^1^H NMR spectrum of **2** at room temperature suggesting a fluxional behaviour in solution of the Cp* ligands bound to Al. Surprisingly, the ^31^P{^1^H} NMR spectrum of **2** showed only two singlets at *δ*=73.4 ppm (*cyclo*‐P_4_(AlCp*)) and *δ*=−202.9 ppm, respectively. No P–P coupling pattern for the *cyclo*‐P_4_(AlCp*) unit was observed even at low temperatures, although a broadening and splitting of the signals was noticed with decreasing temperatures (Figure S11, Supporting Information).

The regioselectivity of the insertion reaction of [Cp*Al^III^]^2+^ in two adjacent P−P bonds may arise by formation of the proposed intermediate **2 i** (Scheme 2). The intermediate **2 i** has an envelope‐shaped *cyclo*‐P_5_ ring, in which the P−P bonds, out of the planar P_4_ fragment η^4^‐coordinated to the [Cp*Fe]^+^ moiety, are the most susceptible for insertion reactions. An NMR‐scale reaction between [(Cp*Al^I^)_4_] and [Cp*Fe(η^5^‐P_5_)] in the presence of dimethoxyethane showed the formation of this possible intermediate (Figures S12,S13). In order to trap such an intermediate, the reaction between [(Cp*Al^I^)_4_] and [Cp*Fe(η^5^‐P_5_)] in a molar ratio of 1:4 was carried out in the presence of 1,3,4,5‐tetramethylimidazolin‐2‐ylidene (ITMe) at 60 °C. As a result, [(Cp*Al^III^ITMe)(μ,η^3^:η^4^‐P_5_)FeCp*] (**3**) was isolated in 63 % yield as a masked intermediate (Scheme 3). In the solid state, **3** forms a carbene stabilized Al–Fe triple‐decker complex analogous to the proposed intermediate **2 i** (Figure 4). The Al centre is η^3^‐coordinated to the cyclo‐P_5_ ring and η^1^‐coordinated to the Cp* ring. The Al−P bond lengths are similar to those in complex **1**. The Al−C1(carbene) (2.017(6) Å) bond length is in line with previous reports.^19^ The ^1^H NMR spectrum of complex **3** (203 K) features only one singlet for the [AlCp*] methyl protons, indicating a fluxional behaviour in solution. The ^31^P{^1^H} NMR spectrum (203 K) showed five sets of multiplets at *δ*(ppm)=−96.2, −58.6, 53.2, 103.6, and 138.7 corresponding to the envelope‐shaped *cyclo*‐P_5_ ring (details in Section 3.5 in the Supporting Information).

**Figure 4:**
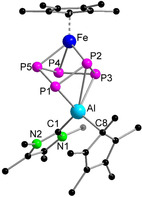
Molecular structure of **3** in the solid state. H atoms are omitted for clarity.

**Scheme 3:**
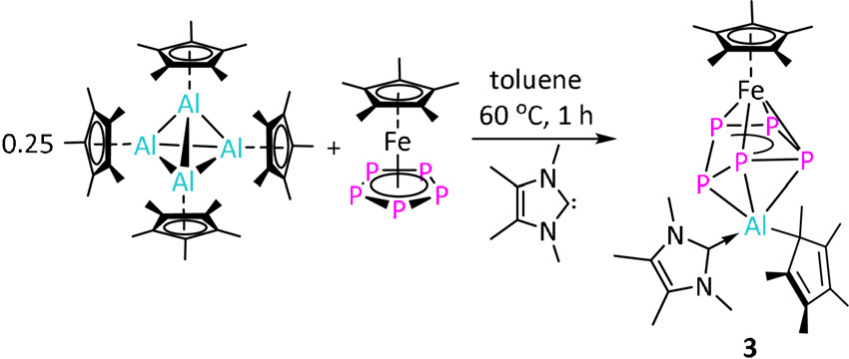
Synthesis of complex **3**.

To obtain a better view in the energetics of the system under discussion, theoretical DFT calculations were performed (technical details are given in the Supporting Information). The results are as follows:

$$\left[\mathrm{Dipp}\text{-}BDI\mathrm{Al}{}^{I}\right]+\left[\mathrm{Cp}{}^{*}\mathrm{Fe}\left(\eta {}^{5}\text{-}P{}_{5}\right)\right]\to \;\left[\left(\mathrm{Dipp}\text{-}BDI\mathrm{Al}{}^{\mathrm{III}}\right)\left(\mu ,\eta {}^{3}:\eta {}^{4}\text{-}P{}_{5}\right)\mathrm{FeCp}{}^{*}\right]\;\left(1\right)\;\Delta E=-115.0\;\mathrm{kJ}\;\mathrm{mol}{}^{-1}$$

$$1/4\left[\left(\mathrm{Cp}{}^{*}\mathrm{Al}{}^{I}\right){}_{4}\right]+\left[\mathrm{Cp}{}^{*}\mathrm{Fe}\left(\eta {}^{5}\text{-}P{}_{5}\right)\right]\to \;\left[\left(\mathrm{Cp}{}^{*}\mathrm{Al}\right)\left(\mu ,\eta {}^{3}:\eta {}^{4}\text{-}P{}_{5}\right)\mathrm{FeCp}{}^{*}\right]\;\left(2\;i\right)\;\Delta E=-33.3\;\mathrm{kJ}\;\mathrm{mol}{}^{-1}$$

as well as

$$\left[\left(\mathrm{Cp}{}^{*}\mathrm{Al}\right)\left(\mu ,\eta {}^{3}:\eta {}^{4}\text{-}P{}_{5}\right)\mathrm{FeCp}{}^{*}\right]\;\left(2\;i\right)+1/2\left[\left(\mathrm{Cp}{}^{*}\mathrm{Al}{}^{I}\right){}_{4}\right]\to \;\left[\left(\mu {}_{3}\text{-}P\right)\left(\mathrm{Cp}{}^{*}\mathrm{Al}{}^{\mathrm{III}}\right){}_{2}\left\{P{}_{4}\left(\mathrm{Al}{}^{\mathrm{III}}\mathrm{Cp}{}^{*}\right)\right\}\left(\mathrm{FeCp}{}^{*}\right)\right]\;\left(2\right)\;\Delta E=-139.6\;\mathrm{kJ}\;\mathrm{mol}{}^{-1}$$

$$1/4\left[\left(\mathrm{Cp}{}^{*}\mathrm{Al}{}^{I}\right){}_{4}\right]+\left[\mathrm{Cp}{}^{*}\mathrm{Fe}\left(\eta {}^{5}\text{-}P{}_{5}\right)\right]+\mathrm{ITMe}\to \;\left[\left(\mathrm{Cp}{}^{*}\mathrm{Al}{}^{\mathrm{III}}\mathrm{ITMe}\right)\left(\mu ,\eta {}^{3}:\eta {}^{4}\text{-}P{}_{5}\right)\mathrm{FeCp}{}^{*}\right]\;\left(3\right)\;\Delta E=-144.8\;\mathrm{kJ}\;\mathrm{mol}{}^{-1}$$

$$1/4\left[\left(\mathrm{Cp}{}^{*}\mathrm{Al}{}^{I}\right){}_{4}\right]\to \left[\mathrm{Cp}{}^{*}\mathrm{Al}{}^{I}\right]\;\Delta E=+29.4\;\mathrm{kJ}\;\mathrm{mol}{}^{-1}$$

Although probably resulting from combined steric and electronic effects, the larger energy gain in the formation of **1**, in contrast to hypothetical **2 i**, is qualitatively made plausible on the basis of the calculated localized MOs with prominent Al−P bonding character (see Figures S31 and S32 in the Supporting Information). For **1**, two Al−P bonds in the range around 2.4 Å are found, while only one Al−P bond is found for **2 i**. Shared electron numbers (SEN) as reliable measures for covalent bonding obtained from Ahlrichs–Heinzmann population analyses^20^ confirm these findings. The reaction step of **2 i** with two equivalent units of [Cp*Al^I^] leading to **2** is highly exothermic (−139.6 kJ mol^−1^) due to the formation of a stable Al/P framework. The high two‐centre SEN(Al‐P) and three‐centre SEN(Al‐Al‐P) confirm this hypothesis and can explain the unexpectedly short Al–Al distances. A comparable situation has been intensively investigated in the compound [P_4_(AlCp*)_6_].^13^ The highly exothermic reaction of hypothetical **2 i** and the carbene ITMe leading to **3** (−111.5 kJ mol^−1^) is explained by substantial electron transfer away from the carbene (0.48 electron) accompanied by the formation of a strong Al−C bond (SEN(Al‐C)=1.04).

In summary, we have studied the reactivity of [Cp*Fe(η^5^‐P_5_)] with monovalent aluminum complexes. In case of monomeric [Dipp‐*BDI*Al^I^], a neutral triple‐decker Al‐Fe polyphosphide complex **1** was obtained. Complex **1** consists of a formally di‐reduced anion [(μ,η^3^:η^4^‐P_5_)FeCp*]^2−^ coordinated to a [Dipp‐*BDI*Al^III^]^2+^ moiety. In contrast, the reaction between tetrameric [(Cp*Al^I^)_4_] and [Cp*Fe(η^5^‐P_5_)] in a molar ratio of 3:4 resulted in an unprecedented Al–Fe polyphosphide cluster **2** containing four metal atoms, which is formed by the regioselective insertion of three [Cp*Al^III^]^2+^ moieties in the *cyclo*‐P_5_ ring of [Cp*Fe(η^5^‐P_5_)]. The formation of intermediate **2 i** could explain the [4+1] fragmentation of the *cyclo*‐P_5_ ring. The possible intermediate for the insertion product was stabilized by using a strong σ‐donor carbene, resulting in the Al–Fe triple‐decker type polyphosphide **3**. Noticeably, these findings highlight the role of supporting ligands and donor groups in the reduction chemistry of polyphosphide systems. In addition, this work establishes a route to directly access Al containing heterometallic polyphosphide complexes.

## Conflict of interest

The authors declare no conflict of interest.

## Supporting information

As a service to our authors and readers, this journal provides supporting information supplied by the authors. Such materials are peer reviewed and may be re‐organized for online delivery, but are not copy‐edited or typeset. Technical support issues arising from supporting information (other than missing files) should be addressed to the authors.

Supplementary

## References

[1a] T. J. Kealy , P. L. Pauson , Nature 1951, 168, 1039;

[1b] G. Wilkinson , M. Rosenblum , M. C. Whiting , R. B. Woodward , J. Am. Chem. Soc. 1952, 74, 2125.

[2a] M. Baudler , Angew. Chem. Int. Ed. Engl. 1982, 21, 492;

[2b] H. Grützmacher , Z. Anorg. Allg. Chem. 2012, 638, 1877.

[3] O. J. Scherer , T. Brück , Angew. Chem. Int. Ed. Engl. 1987, 26, 59;

[4a] J. Bai , A. V. Virovets , M. Scheer , Science 2003, 300, 781; 12730597 10.1126/science.1081119

[4b] M. Scheer , L. J. Gregoriades , A. V. Virovets , W. Kunz , R. Neueder , I. Krossing , Angew. Chem. Int. Ed. 2006, 45, 5689; 10.1002/anie.200601546 16862629

[4c] M. Scheer , A. Schindler , R. Merkle , B. P. Johnson , M. Linseis , R. Winter , C. E. Anson , A. V. Virovets , J. Am. Chem. Soc. 2007, 129, 13386; 17929817 10.1021/ja075926m

[4d] M. Scheer , Dalton Trans. 2008, 4372; 18698438 10.1039/b718179p

[4e] F. Dielmann , M. Fleischmann , C. Heindl , E. V. Peresypkina , A. V. Virovets , R. M. Gschwind , M. Scheer , Chem. Eur. J. 2015, 21, 6208. 25759976 10.1002/chem.201500692 PMC4464546

[5a] T. Li , J. Wiecko , N. A. Pushkarevsky , M. T. Gamer , R. Köppe , S. N. Konchenko , M. Scheer , P. W. Roesky , Angew. Chem. Int. Ed. 2011, 50, 9491; 10.1002/anie.201102748 21882304

[5b] M. V. Butovskiy , G. Balázs , M. Bodensteiner , E. V. Peresypkina , A. V. Virovets , J. Sutter , M. Scheer , Angew. Chem. Int. Ed. 2013, 52, 2972; 10.1002/anie.201209329 23364883

[5c] T. Li , M. T. Gamer , M. Scheer , S. N. Konchenko , P. W. Roesky , Chem. Commun. 2013, 49, 2183; 10.1039/c3cc38841g 23385547

[5d] E. Mädl , M. V. Butovskii , G. Balázs , E. V. Peresypkina , A. V. Virovets , M. Seidl , M. Scheer , Angew. Chem. Int. Ed. 2014, 53, 7643; 10.1002/anie.201403581 24895298

[5e] C. Schoo , S. Bestgen , M. Schmidt , S. N. Konchenko , M. Scheer , P. W. Roesky , Chem. Commun. 2016, 52, 13217. 10.1039/c6cc07367k 27738672

[6] R. F. Winter , W. E. Geiger , Organometallics 1999, 18, 1827.

[7] A. E. Seitz , F. Hippauf , W. Kremer , S. Kaskel , M. Scheer , Nat. Commun. 2018, 9, 361. 29367623 10.1038/s41467-017-02735-2 PMC5783940

[8] R. Yadav , T. Simler , S. Reichl , B. Goswami , C. Schoo , R. Köppe , M. Scheer , P. W. Roesky , J. Am. Chem. Soc. 2020, 142, 1190. 31860286 10.1021/jacs.9b12151

[9a] C. Dohmeier , C. Robl , M. Tacke , H. Schnöckel , Angew. Chem. Int. Ed. Engl. 1991, 30, 564;

[9b] S. Schulz , H. W. Roesky , H. J. Koch , G. M. Sheldrick , D. Stalke , A. Kuhn , Angew. Chem. Int. Ed. Engl. 1993, 32, 1729;

[9c] J. Gauss , U. Schneider , R. Ahlrichs , C. Dohmeier , H. Schnoeckel , J. Am. Chem. Soc. 1993, 115, 2402;

[9d] C. Dohmeier , H. Krautscheid , H. Schnöckel , Angew. Chem. Int. Ed. Engl. 1995, 33, 2482;

[9e] C. Dohmeier , D. Loos , H. Schnöckel , Angew. Chem. Int. Ed. Engl. 1996, 35, 129;

[9f] A. Purath , C. Dohmeier , A. Ecker , H. Schnöckel , K. Amelunxen , T. Passler , N. Wiberg , Organometallics 1998, 17, 1894;

[9g] C. Schnitter , H. W. Roesky , C. Röpken , R. Herbst-Irmer , H.-G. Schmidt , M. Noltemeyer , Angew. Chem. Int. Ed. 1998, 37, 1952;

[9h] H. Sitzmann , M. F. Lappert , C. Dohmeier , C. Üffing , H. Schnöckel , J. Organomet. Chem. 1998, 561, 203;

[9i] A. Purath , R. Köppe , H. Schnöckel , Angew. Chem. Int. Ed. 1999, 38, 2926; 10.1002/(sici)1521-3773(19991004)38:19<2926::aid-anie2926>3.0.co;2-b 10540395

[9j] C. Cui , H. W. Roesky , H.-G. Schmidt , M. Noltemeyer , H. Hao , F. Cimpoesu , Angew. Chem. Int. Ed. 2000, 39, 4274; 10.1002/1521-3773(20001201)39:23<4274::AID-ANIE4274>3.0.CO;2-K 29711904

[9k] M. Schormann , K. S. Klimek , H. Hatop , S. P. Varkey , H. W. Roesky , C. Lehmann , C. Röpken , R. Herbst-Irmer , M. Noltemeyer , J. Solid State Chem. 2001, 162, 225;

[9l] H. W. Roesky , S. S. Kumar , Chem. Commun. 2005, 4027. 10.1039/b505307b 16091791

[10a] J. Hicks , P. Vasko , J. M. Goicoechea , S. Aldridge , Nature 2018, 557, 92; 29662211 10.1038/s41586-018-0037-y

[10b] A. Hofmann , T. Tröster , T. Kupfer , H. Braunschweig , Chem. Sci. 2019, 10, 3421. 30996931 10.1039/c8sc05175e PMC6429597

[11a] H. W. Roesky , Inorg. Chem. 2004, 43, 7284; 15530077 10.1021/ic0400641

[11b] R. J. Wright , M. Brynda , P. P. Power , Angew. Chem. Int. Ed. 2006, 45, 5953; 10.1002/anie.200601925 16897794

[11c] P. Bag , C. Weetman , S. Inoue , Angew. Chem. Int. Ed. 2018, 57, 14394; 10.1002/anie.201803900 29790227

[11d] T. Chu , G. I. Nikonov , Chem. Rev. 2018, 118, 3608; 29558125 10.1021/acs.chemrev.7b00572

[11e] S. J. Urwin , G. S. Nichol , M. J. Cowley , Chem. Commun. 2018, 54, 378; 10.1039/c7cc08415c 29242890

[11f] V. Nesterov , D. Reiter , P. Bag , P. Frisch , R. Holzner , A. Porzelt , S. Inoue , Chem. Rev. 2018, 118, 9678. 29969239 10.1021/acs.chemrev.8b00079

[12a] Y. Peng , H. Fan , H. Zhu , H. W. Roesky , J. Magull , C. E. Hughes , Angew. Chem. Int. Ed. 2004, 43, 3443; 10.1002/anie.200353406 15221834

[12b] A. C. Stelzer , P. Hrobárik , T. Braun , M. Kaupp , B. Braun-Cula , Inorg. Chem. 2016, 55, 4915. 27129027 10.1021/acs.inorgchem.6b00462

[13] C. Dohmeier , H. Schnöckel , C. Robl , U. Schneider , R. Ahlrichs , Angew. Chem. Int. Ed. Engl. 1994, 33, 199;

[14] S. González-Gallardo , T. Bollermann , R. A. Fischer , R. Murugavel , Chem. Rev. 2012, 112, 3136. 22364369 10.1021/cr2001146

[15] Y. Liu , J. Li , X. Ma , Z. Yang , H. W. Roesky , Coord. Chem. Rev. 2018, 374, 387.

[16] P. Pyykkö , M. Atsumi , Chem. Eur. J. 2009, 15, 12770. 19856342 10.1002/chem.200901472

[17a] S. Welsch , L. J. Gregoriades , M. Sierka , M. Zabel , A. V. Virovets , M. Scheer , Angew. Chem. Int. Ed. 2007, 46, 9323; 10.1002/anie.200704015 17972256

[17b] M. Fleischmann , S. Welsch , H. Krauss , M. Schmidt , M. Bodensteiner , E. V. Peresypkina , M. Sierka , C. Gröger , M. Scheer , Chem. Eur. J. 2014, 20, 3759. 24615817 10.1002/chem.201304466

[18a] V. Miluykov , A. Kataev , O. Sinyashin , P. Lönnecke , E. Hey-Hawkins , Organometallics 2005, 24, 2233;

[18b] C. M. Hoidn , T. M. Maier , K. Trabitsch , J. J. Weigand , R. Wolf , Angew. Chem. Int. Ed. 2019, 58, 18931; 10.1002/anie.201908744 PMC6972699 31573718

[19] P. Bag , A. Porzelt , P. J. Altmann , S. Inoue , J. Am. Chem. Soc. 2017, 139, 14384. 28898060 10.1021/jacs.7b08890

[20] R. Heinzmann , R. Ahlrichs , Theor. Chim. Acta 1976, 42, 33.

