# Supplementary material for: Regioselective Insertion of Aluminum(I) in the cyclo‐P5 Ring of Pentaphosphaferrocene
Source: Angew Chem Int Ed Engl. 2020 Apr 19;59(24):9443–7. doi: 10.1002/anie.202002774 (PMC7318284; doi:10.1002/anie.202002774)
Supplement: Supplementary file 1 — Supplementary [file ANIE-59-9443-s001.pdf]

Angewandte Chemie
Eine Zeitschrift der Gesellschaft Deutscher Chemiker

Supporting Information

Regioselective Insertion of Aluminum(I) in the *cyclo*-P_5 Ring of Pentaphosphaferrocene

*Ravi Yadav, Thomas Simler, Bhupendra Goswami, Christoph Schoo, Ralf Köppe, Subhayan Dey, and Peter W. Roesky**

anie_202002774_sm_miscellaneous_information.pdf

Contents

1 Experimental section S2

1.1 General methods S2

1.2 Synthesis of [(Dipp-*BDI*Al^{III})(μ-η^3-η^4-P_5)FeCp*] (**1**) S2

1.3 Synthesis of [(μ_3-P)(Cp*Al^{III})$_2${P_4(Al^{III}Cp*)}(FeCp*)] (**2**) S3

1.4 Synthesis of [(Cp*Al^{III}ITMe)(μ-η^3-η^4-P_5)FeCp*] (**3**) S3

2 NMR Spectra S5

3 Details of the simulations of the $^{31}P\{^1H\}$ NMR spectra. S15

3.1 Experimental and simulated spectra of **1** at 233 K. S15

3.2 Table S1. Chemical shifts, coupling constants and linewidths from the iterative fit of the AA'MXX' spin system of **1** at 298 K. S17

3.3 Experimental and simulated spectra of **2i** at 298 K S18

3.4 Table S2. Chemical shifts, coupling constants and linewidths from the iterative fit of the AMM'XX' spin system of **2i** at 298 K. S20

3.5 Experimental and simulated spectra of **3** at 203 K S21

3.6 Table S3. Chemical shifts, coupling constants and linewidths from the iterative fit of the P_5 spin system of complex **3** in toluene-*d*$_8$ at 203 K. S23

4 IR Spectra S24

5 Single crystal X-ray diffraction S26

5.1 Table S4. Crystal data and structure refinement S27

5.2 Crystal Structures S28

6. Quantum Chemical Calculations S31

7 References S52

1 Experimental section

1.1 General methods

All the manipulations of air- and water-sensitive reactions were performed with rigorous exclusion of oxygen and moisture in flame-dried Schlenk-type glassware either on a dual manifold Schlenk line, interfaced to a high vacuum (10^{-3} torr) line or in an argon-filled MBraun glove box. Solvents were dried by using an MBraun solvent purification system (SPS 800), degassed and stored *in vacuo* over $LiAlH_4$. Elemental analyses were carried out with an Elementar vario Micro cube. Toluene-d_8 and C_6D_6 were stored over Na/K alloy and were degassed by freeze-pump-thaw cycles. IR spectra were obtained on a Bruker Tensor 37 spectrometer equipped with a room temperature DLaTGS detector and a diamond ATR (attenuated total reflection) unit. NMR spectra were recorded on Bruker spectrometers (Avance III 400 MHz). Chemical shifts are referenced using signals of the residual protio solvent (^{1}H) or the solvent (^{13}C{^{1}H}) and are reported relative to tetramethylsilane. The chemical shifts in the ^{31}P{^{1}H} spectra are reported relative to H_3PO_4 (85%). All NMR spectra were measured at 298 K, unless otherwise specified. The multiplicity of the signals is indicated as s = singlet, d = doublet, t = triplet, sept = septet, m = multiplet and br = broad. [Dipp-*BDI*Al^I][1] (Dipp-*BDI* = {[2,6-^{i}Pr$_2$C$_6$H$_3$NCMe]$_2$CH}$^-$), [(Cp*Al^I)$_4$][2] (Cp* = C_5Me_5), [Cp*Fe(η^5-P$_5$)][3] , and 1,3,4,5-tetramethylimidazolin-2-ylidene[4] (ITMe) were prepared according to the literature procedures.

1.2 Synthesis of [(Dipp-*BDI*Al^{III})(μ-η^3-η^4-P$_5$)FeCp*] (1)

A solution of [Dipp-*BDI*Al^I] (50 mg, 0.112 mmol) in toluene (5 mL) was added to a solution of [Cp*Fe(η^5-P$_5$)] (39 mg, 0.112 mmol) in toluene (5 mL) at -78 °C. The reaction mixture was allowed to warm up to room temperature and stirred for 1 hour. It was then transferred into a double ampoule which was afterwards flame sealed *in vacuo*. Single crystals were grown by slow evaporation of the toluene solvent. The mother liquor was decanted off and the crystals were dried under vacuum. Yield = 31 mg (based on crystals), 0.039 mmol, 35%. Anal. Calcd. for $C_{39}H_{56}N_2P_5AlFe$ (790.59): C, 59.25; H, 7.14; N, 3.54. Found: C, 59.70; H, 7.38; N, 3.20.

^{1}H NMR (400 MHz, 298 K, C_6D_6): δ [ppm] = 1.04 (br, 12 H, CH(C*H*$_3$)$_2$, $\Delta\nu_{1/2}$ ≈ 16 Hz), 1.22 (s, 15 H, C(C*H*$_3$) Cp*), 1.44 (s, 6 H, NC(C*H*$_3$)), 1.93 (d, 12 H, CH(C*H*$_3$)$_2$, $^3J_{HH}$ = 6.6 Hz), 3.37 (m, 4 H, C*H*(CH$_3$)), 4.71 (s, 1 H, NC(C*H*)CN), 7.29-7.35 (m, 6 H, Dipp-C*H*$_{arom}$).

^{31}**P{^{1}H} NMR** (162 MHz, 298 K, C_6D_6): δ[ppm] = 32.1 (br, $\Delta\nu_{1/2}$ ≈ 400 Hz), 60.7 (br, $\Delta\nu_{1/2}$ ≈ 400 Hz), 98.4 (br, $\Delta\nu_{1/2}$ ≈ 400 Hz).

^{31}**P{^{1}H} NMR** (162 MHz, 233 K, toluene-d_8): δ[ppm] = 30.7 (m, 2 P, $P_{AA'}$), 60.6 (m, 1 P, P_M), and 97.2 (m, 2 P, $P_{XX'}$) (See figures S19, S20 and table S1 for details).

^{13}**C{^{1}H} NMR** (100 MHz, 298 K, C_6D_6): δ[ppm] = 10.4 (C(*C*H$_3$)), 25.3 (*C*H$_3$CCHC*C*H$_3$), 25.3 (CH(*C*H$_3$)$_2$), 25.4 (CH(*C*H$_3$)$_2$), 29.3 (*C*H(CH$_3$)$_2$), 92.5 (*C*(CH$_3$)), 98.0 (CH$_3$*C*CHCCH$_3$), 125.1 (Dipp-C_{arom}), 128.6 (Dipp-C_{arom}), 128.7 (Dipp-C_{arom}), 129.3 (Dipp-C_{arom}), 141.6 (Dipp-C_{arom}), 172.0 (*C*N).

1.3 Synthesis of [(μ_3-P)(Cp*Al^{III})$_2${P$_4$(Al^{III}Cp*)}(FeCp*)] (2)

Toluene (15 mL) was condensed onto a mixture of [(Cp*Al^I)$_4$] (140 mg, 0.216 mmol) and [Cp*Fe(η^5-P$_5$)] (100 mg, 0.289 mmol) at -78 °C and the reaction mixture was heated in a sealed Young flask to 80 °C for one week. After cooling to room temperature, the reaction mixture was filtered into a double ampoule which was afterwards flame sealed *in vacuo*. Single crystals were grown by slow evaporation of the toluene solvent. The mother liquor was decanted off and the crystals were washed with cold toluene and dried under vacuum. Yield = 114 mg (based on crystals), 0.137 mmol, 47%. Anal. Calcd. for $C_{40}H_{60}P_5Al_3Fe$ (832.58): C, 57.71; H, 7.29. Found: C, 57.66; H, 7.23.

^{1}**H NMR** (400 MHz, 298 K, C_6D_6): δ[ppm] = 1.31 (s, 15 H, C(C*H*$_3$), [Cp*Fe]), 2.17 (s, 45 H, C(C*H*$_3$), [Cp*Al]).

^{31}**P{^{1}H} NMR** (162 MHz, 298 K, C_6D_6): δ[ppm] = -203.5 (s, 1 P), 73.8 (br, 4 P, $\Delta\nu_{1/2}$ ≈ 110 Hz).

^{13}**C{^{1}H} NMR** (100 MHz, 298 K, C_6D_6): δ[ppm] = 10.4 (C(*C*H$_3$), [Cp*Fe]), 12.6 (C(*C*H$_3$), [Cp*Al]), 92.6 ((*C*(CH$_3$), [Cp*Fe]), 117.7 ((*C*(CH$_3$), [Cp*Al]).

1.4 Synthesis of [(Cp*Al^{III}ITMe)(μ-η^3-η^4-P$_5$)FeCp*] (3)

Toluene (15 mL) was condensed onto a mixture of [(Cp*Al^I)$_4$] (50 mg, 0.077 mmol), ITMe (38 mg, 0.308 mmol) [Cp*Fe(η^5-P$_5$)] (106 mg, 0.306 mmol) at -78 °C and the reaction mixture was warmed to room temperature. Subsequently, the reaction mixture was stirred at 60 °C for one hour. After cooling to room temperature, the reaction mixture was filtered, and the filtrate was concentrated till incipient crystallization. The concentrated reaction mixture was stored at -30 °C for one week to obtain a copious amount of crystals. The mother liquor was decanted off and the crystals were washed with pentane (3 x 5 mL) and dried under vacuum. Yield = 123 mg (based on crystals), 0.194 mmol, 63%. Anal. Calcd. for $C_{27}H_{42}P_5N_2AlFe$ (632.34): C, 51.29; H, 6.70; N, 4.43. Found: C, 51.52; H, 6.34; N, 4.38.

^{1}H NMR (400 MHz, 298 K, toluene-d_8): δ [ppm] = 1.19 (s, 6 H, CNC(C*H*$_3$)), 1.51 (s, 15 H, C(C*H*$_3$), [Cp*Fe]), 1.78 (s, 15 H, C(C*H*$_3$), [Cp*Al]), 3.04 (br, 6 H, N(C*H*$_3$), $\Delta\nu_{1/2} \approx$ 70 Hz).

^{1}H NMR (400 MHz, 203 K, toluene-d_8): δ [ppm] = 0.80 (s, 3 H, CNC(C*H*$_3$)), 1.05 (s, 3 H, CNC(C*H*$_3$)), 1.56 (s, 15 H, C(C*H*$_3$), [Cp*Fe]), 1.89 (s, 15 H, C(C*H*$_3$), [Cp*Al]), 2.42 (s, 3 H, N(C*H*$_3$)), 3.36 (s, 3 H, N(C*H*$_3$)).

^{31}P{^{1}H} NMR (162 MHz, 298 K, toluene-d_8): δ [ppm] = -80.2 (br, 1P, $\Delta\nu_{1/2} \approx$ 1200 Hz), -43.8 (br, 1P, $\Delta\nu_{1/2} \approx$ 1200 Hz), 56.3 (br, 1P, $\Delta\nu_{1/2} \approx$ 1000 Hz), 94.8 (br, 1P, $\Delta\nu_{1/2} \approx$ 1000 Hz), 130.4 (br, 1P, $\Delta\nu_{1/2} \approx$ 800 Hz).

^{31}P{^{1}H} NMR (162 MHz, 203 K, toluene-d_8): -96.2 (m, 1 P, P_5), -58.6 (m, 1 P, P_4), 53.2 (m, 1 P, P_3), 103.6 (m, 1 P, P_2), 138.7 (m, 1 P, P_1) (See figures S23, S24, and table S3 for details).

^{13}C{^{1}H} NMR (100 MHz, 298 K, toluene-d_8): δ [ppm] = 7.9 (CNC(*C*H$_3$)), 11.1 (C(*C*H$_3$), [Cp*Fe]), 12.8 (C(*C*H$_3$), [Cp*Al]), 52.2 (N(*C*H$_3$)), 92.9 (*C*(CH$_3$), [Cp*Fe]), 119.1 (*C*(CH$_3$), [Cp*Al]), 124.4 (N*CC*N), N*C*N could not be observed.

2 NMR Spectra

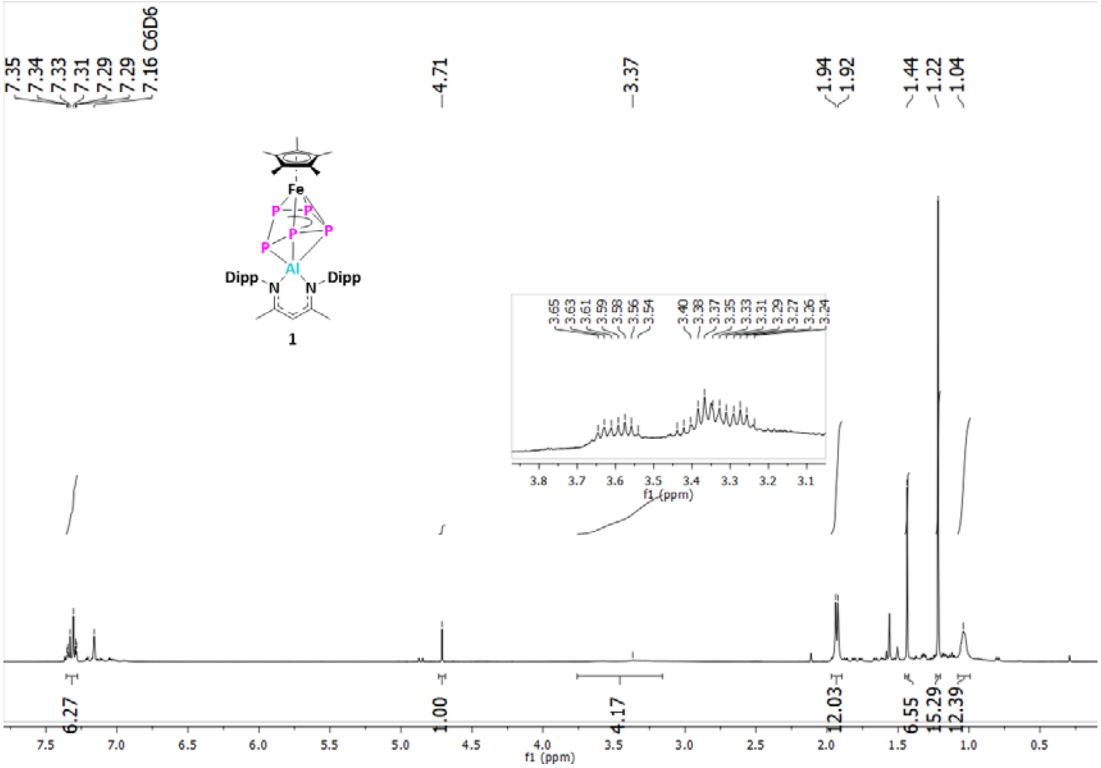

Figure S1. ^{1}H NMR (400 MHz, 298 K, C_6D_6) spectrum of complex **1**.

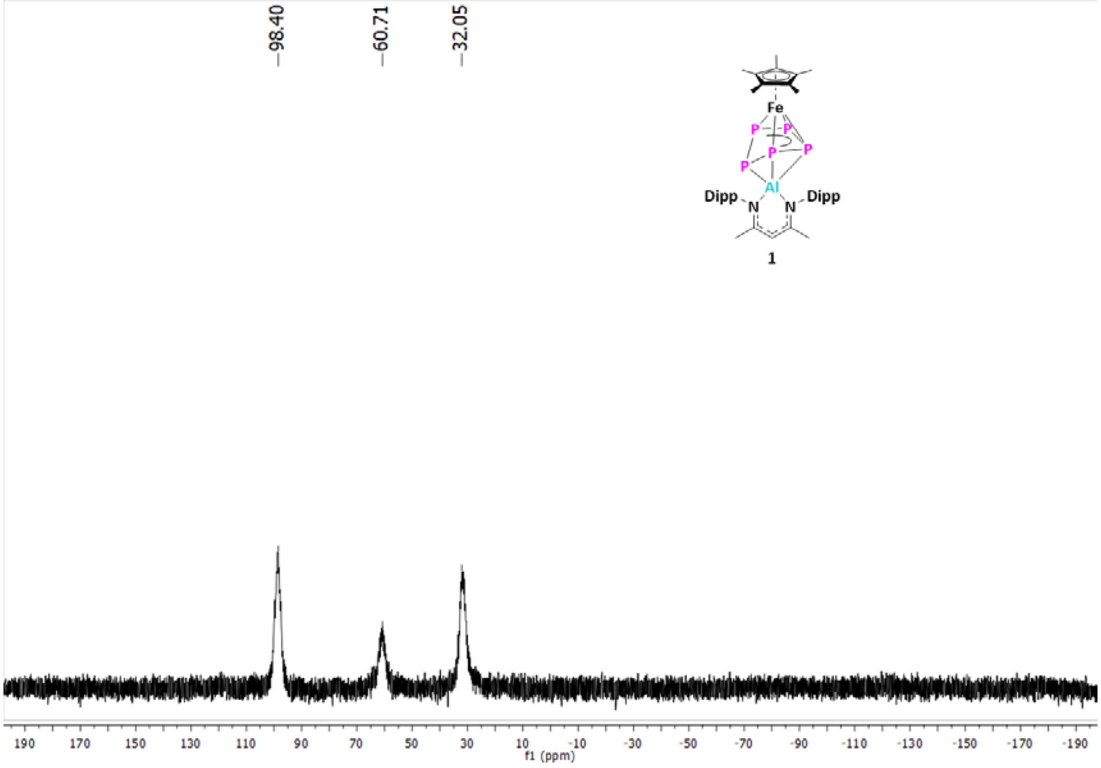

Figure S2. $^{31}P\{^1H\}$ NMR (162 MHz, 298 K, C_6D_6) spectrum of complex **1**.

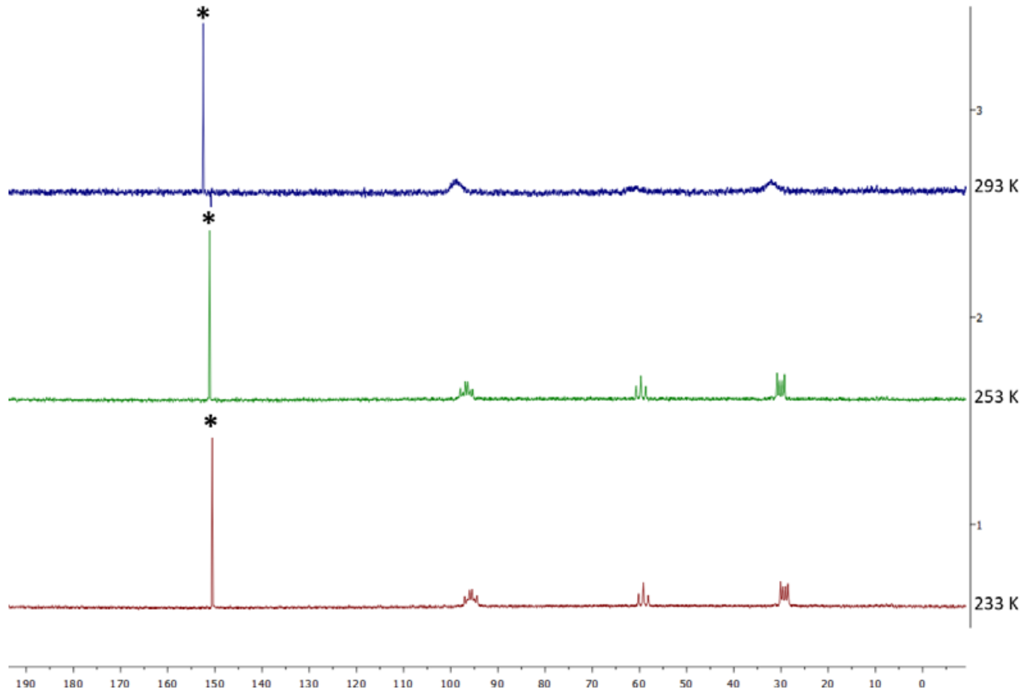

Figure S3. Variable temperature $^{31}P\{^1H\}$ NMR (162 MHz, toluene-d_8) spectra of complex **1**. * = [Cp*Fe(η^5-P_5)].

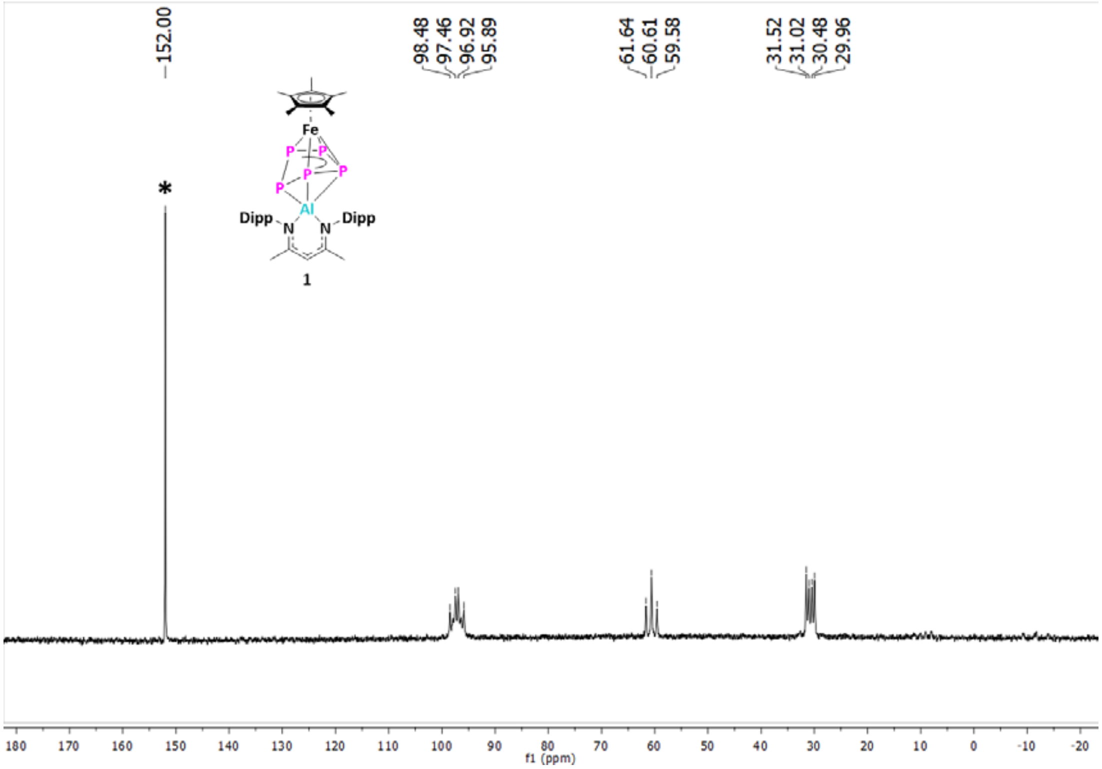

Figure S4. $^{31}P\{^1H\}$ NMR (162 MHz, 233 K, toluene-d_8) spectrum of complex **1**. * = [Cp*Fe(η^5-P_5)].

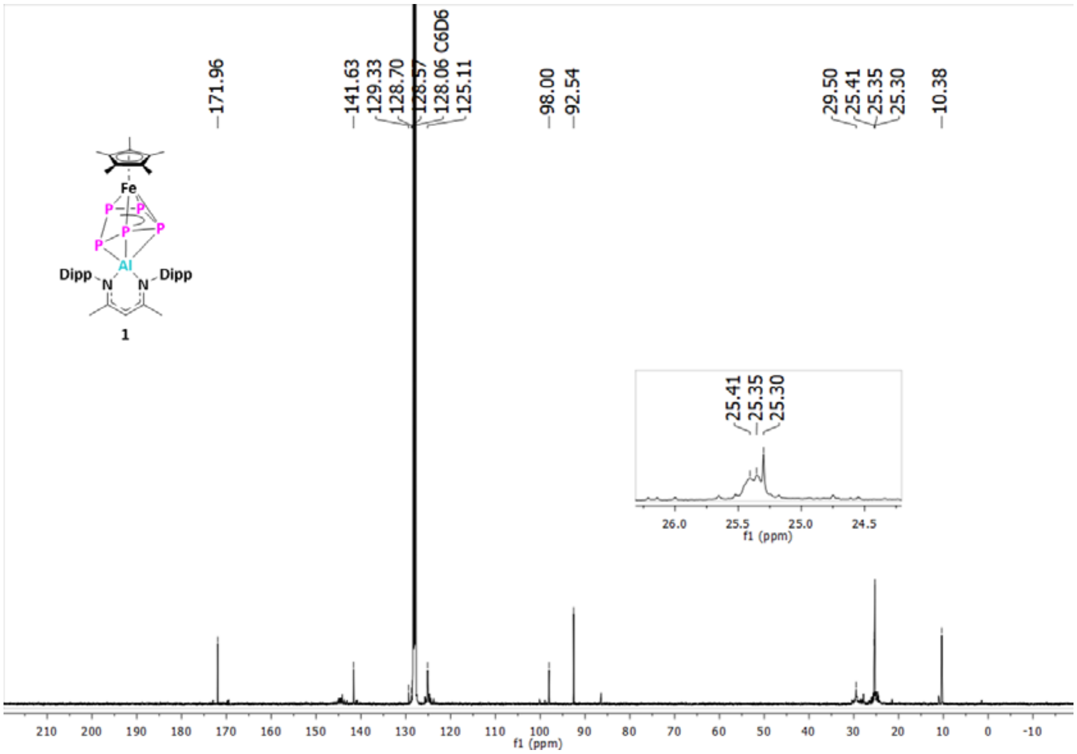

Figure S5. $^{13}C\{^1H\}$ NMR (100 MHz, 298 K, C_6D_6) of complex **1**.

An NMR study revealed that complex **1** further reacts with another equivalent of [Dipp-*BDI*Al^I]. The reaction between [Dipp-*BDI*Al^I] and [Cp*Fe(η^5-P_5)] was carried out in a 2:1 molar ratio, respectively, resulting in the formation of a new species with distinct NMR features (Figures S6 and S7). Unfortunately, despite several attempts, no crystals suitable for X-ray diffraction studies could not be obtained and the exact identity of this product remains unknown.

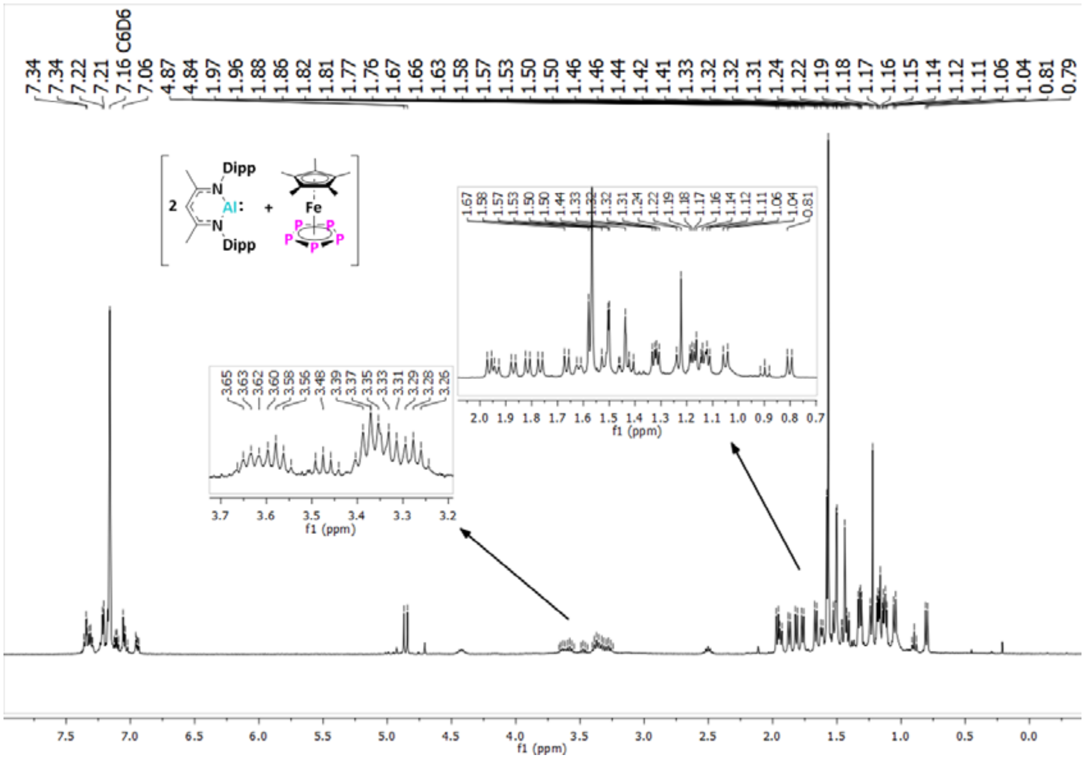

Figure S6. ^{1}H NMR (400 MHz, 298 K, C_6D_6) spectrum of the reaction between [Dipp-*BDI*Al^{I}] and [Cp*Fe(η^5-P_5)] in a 2:1 molar ratio, respectively.

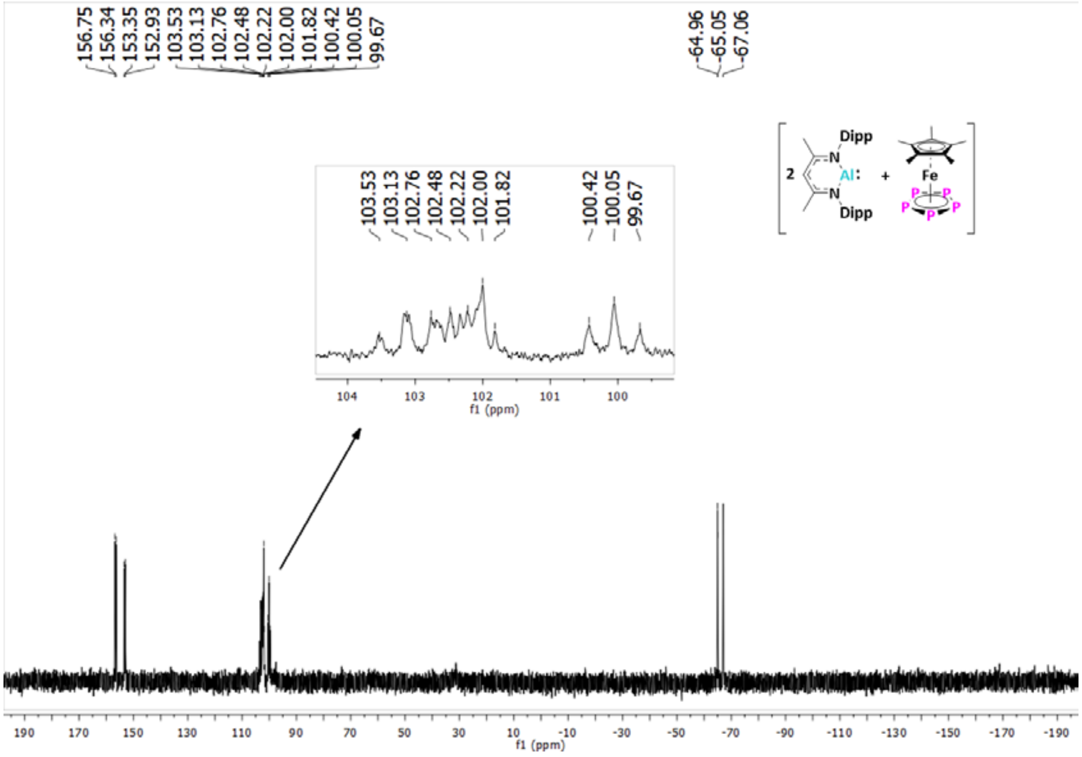

Figure S7. $^{31}P\{^{1}H\}$ NMR (162 MHz, 298 K, C_6D_6) spectrum of the reaction between [Dipp-*BDI*Al^{I}] and [Cp*Fe(η^5-P_5)] in a 2:1 ratio, respectively.

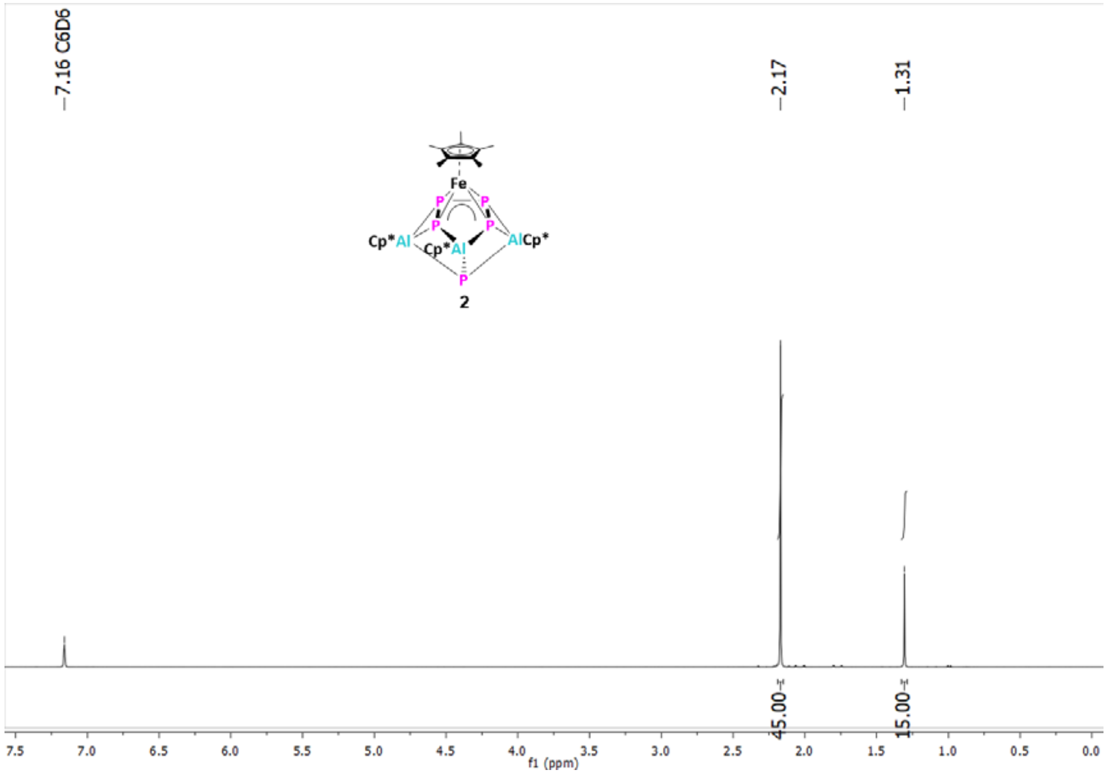

Figure S8. ^{1}H NMR (400 MHz, 298 K, C_6D_6) spectrum of complex **2**.

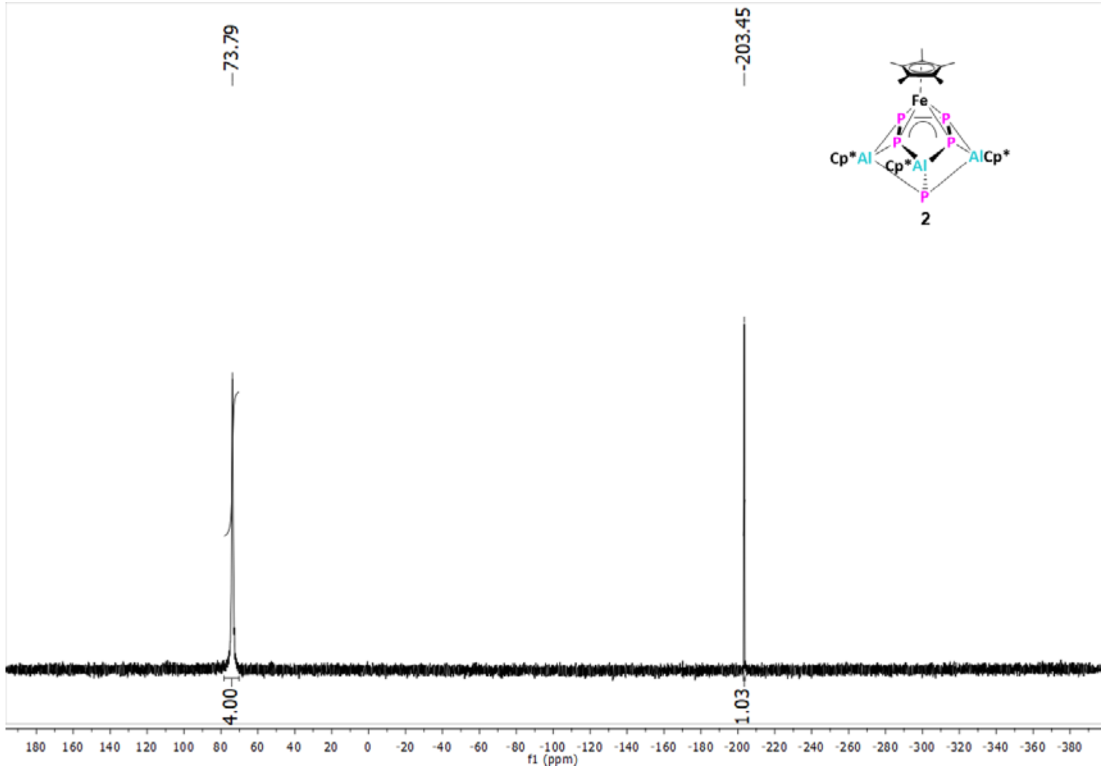

Figure S9. $^{31}P\{^{1}H\}$ NMR (162 MHz, 298 K, C_6D_6) spectrum of complex **2**.

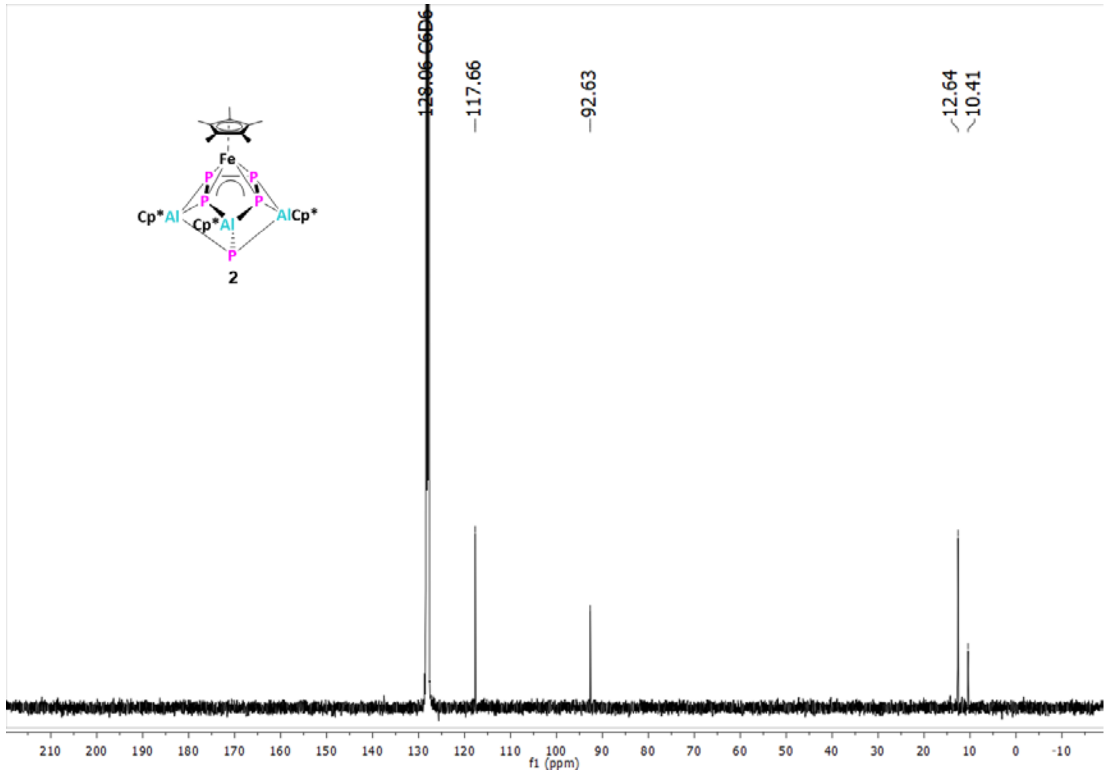

Figure S10. $^{13}C\{^{1}H\}$ NMR (100 MHz, 298 K, C_6D_6) of complex **2**.

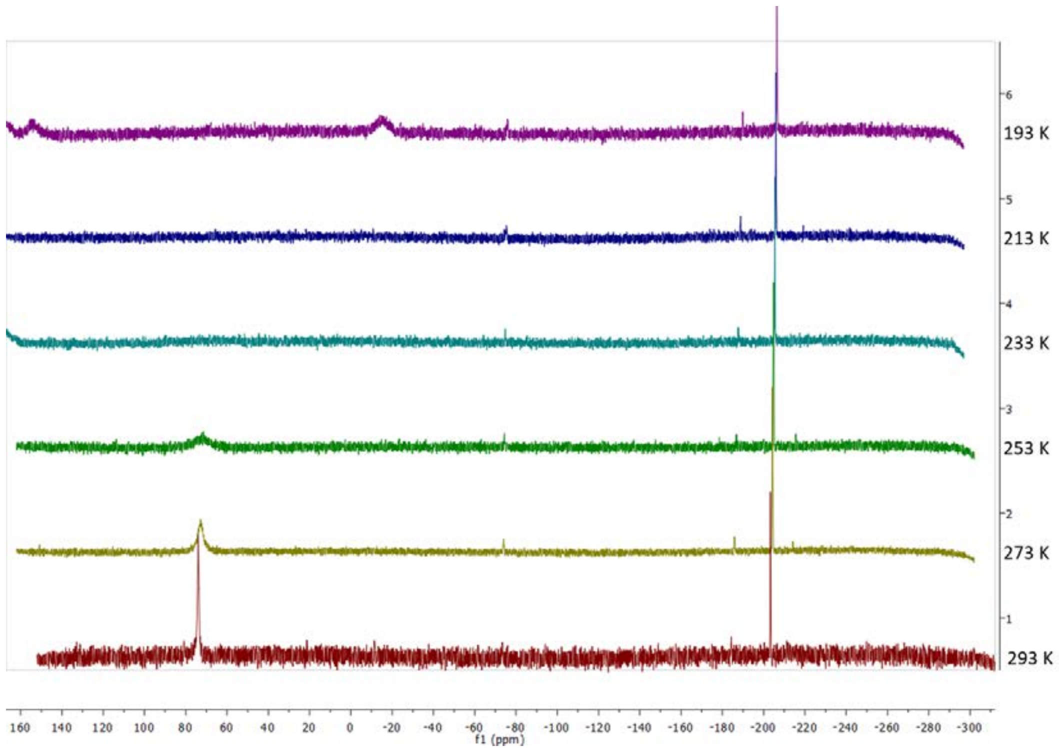

Figure S11. Variable temperature $^{31}P\{^{1}H\}$ NMR (162 MHz, toluene-d_8) spectra of complex **2**.

Trapping of the intermediate in the formation of complex 2:

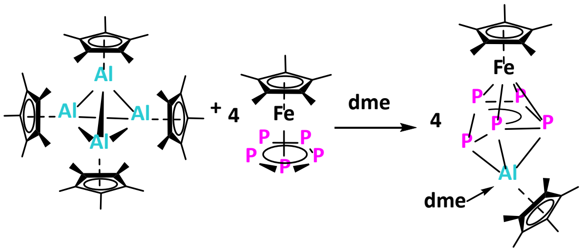

2i(dme)

Scheme S1. The reaction of [(Cp*Al^{I})$_4$] and [Cp*Fe(η^5-P$_5$)] in dme.

An NMR scale reaction between [(Cp*Al^{I})$_4$] and [Cp*Fe(η^5-P$_5$)] in a 3:4 molar ratio, respectively, was carried out in the presence of dimethoxyethane (dme) as a donor ligand (Scheme S1). Initially, a set of three resonances at δ-122.8, 33.9, and 121.3 ppm was observed in the ^{31}P{^{1}H} NMR spectrum, which may correspond to a possible reaction intermediate with coordinated dme (**2i**(dme)) (Figure S12). These signals disappear after a few hours at room temperature, giving rise to the characteristic signals of complex **2** (Figure S13). The ^{31}P{^{1}H} NMR spectrum of **2i**(dme) revealed the presence of an AA'MM'X spin system, which is a typical pattern observed for di-reduced and bent *cyclo*-P$_5$ rings (Figures S21 and S22).

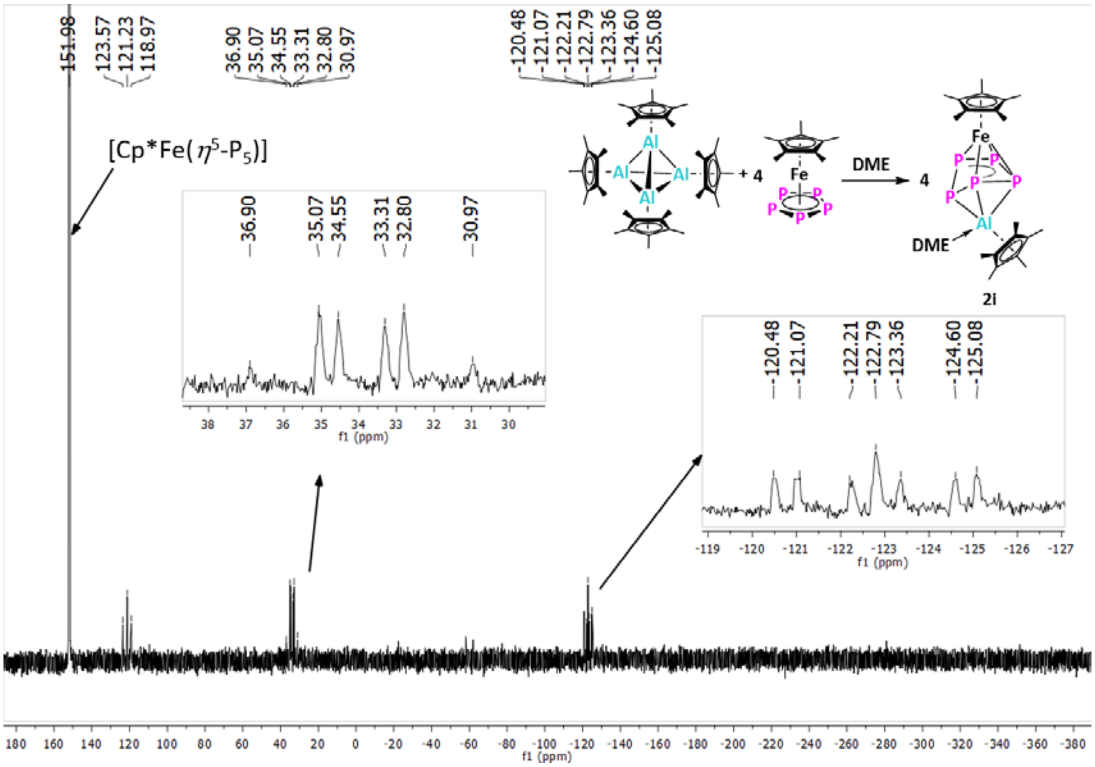

Figure S12. ^{31}P{^{1}H} NMR (162 MHz, 298 K, C$_6$D$_6$ insert in dme) spectrum of the possible intermediate **2i** trapped in dme. The intermediate slowly converts into complex **2**.

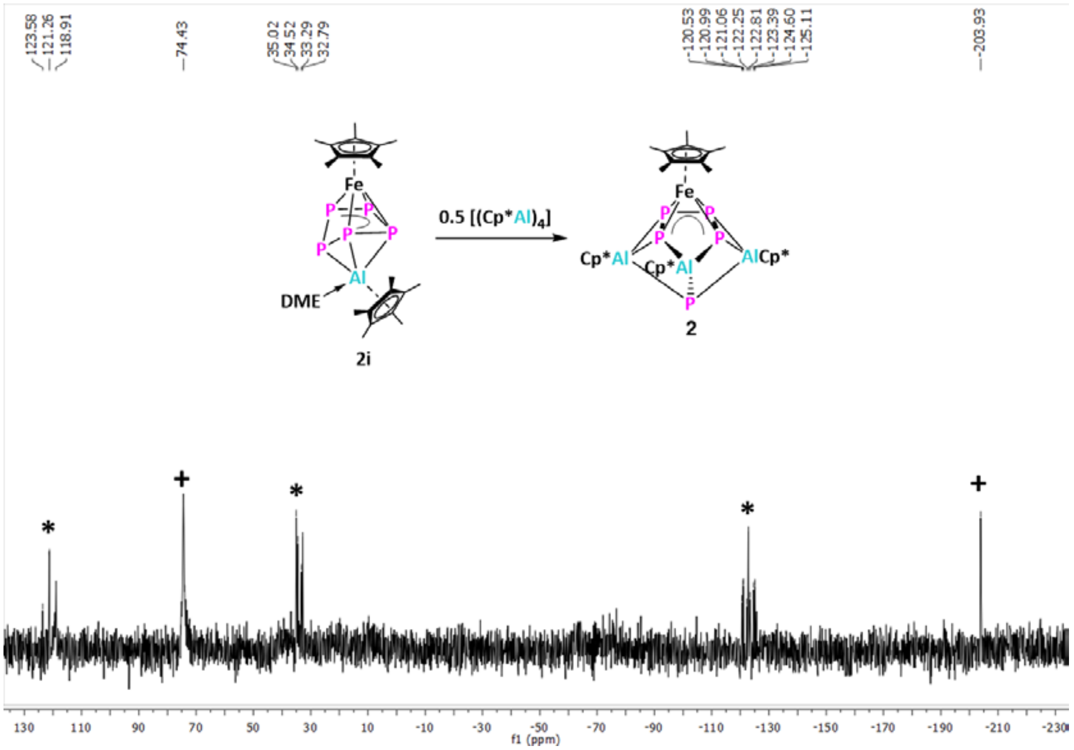

Figure S13. $^{31}P\{^{1}H\}$ NMR (162 MHz, 298 K, C_6D_6 insert in dme) spectrum showing both **2i**(dme) (*) and **2** (+). The intermediate **2i**(dme) slowly converts into complex **2**.

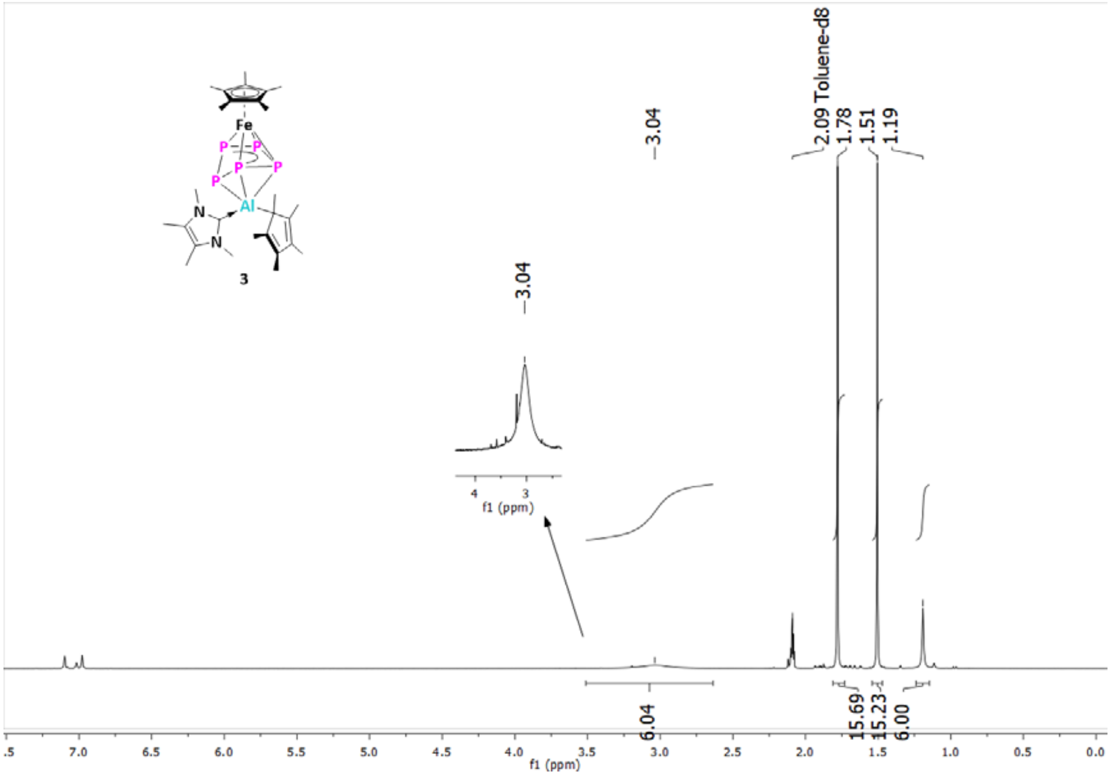

Figure S14. ^{1}H NMR (400 MHz, 298 K, toluene-d_8) spectrum of complex **3**.

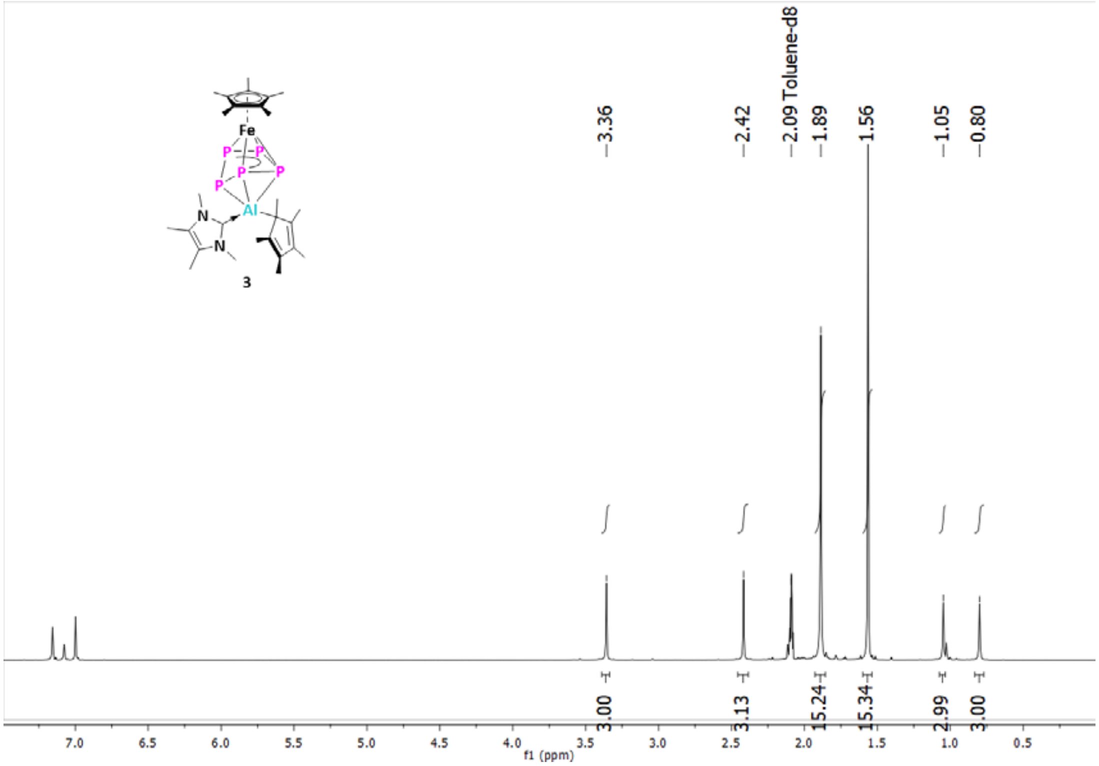

Figure S15. ^{1}H NMR (400 MHz, 203 K, toluene-d_8) spectrum of complex **3**.

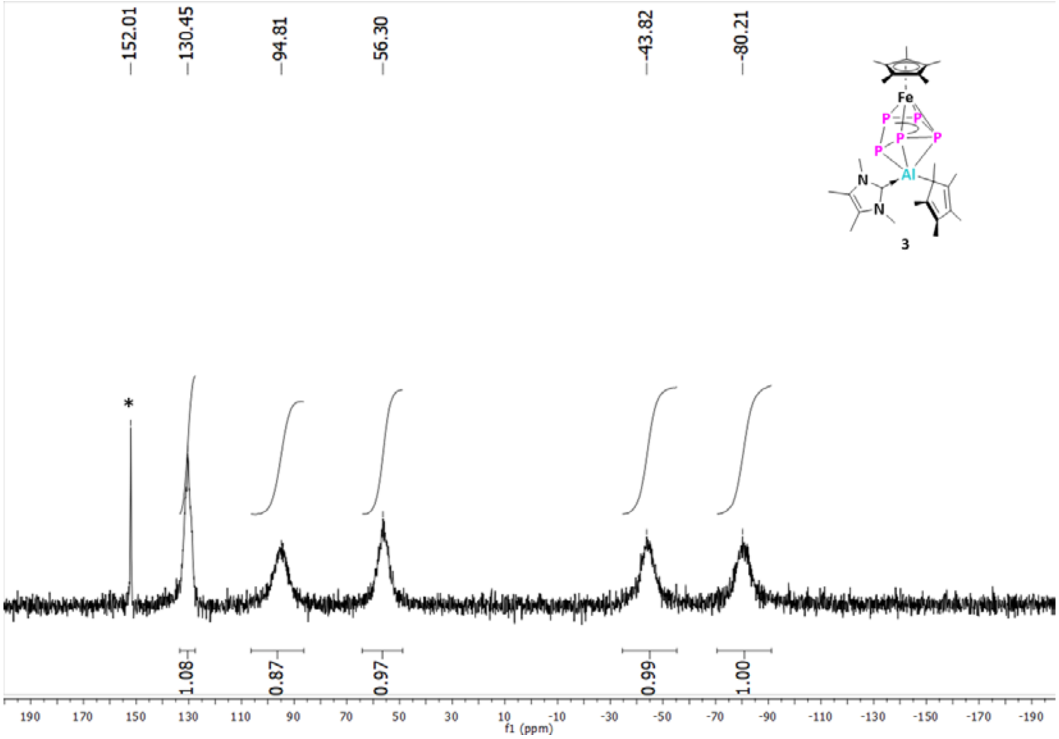

Figure S16. ^{31}P{^{1}H} NMR (162 MHz, 298 K, toluene-d_8) spectrum of complex **3**. (*) = traces of [Cp*Fe(η^5-P_5)].

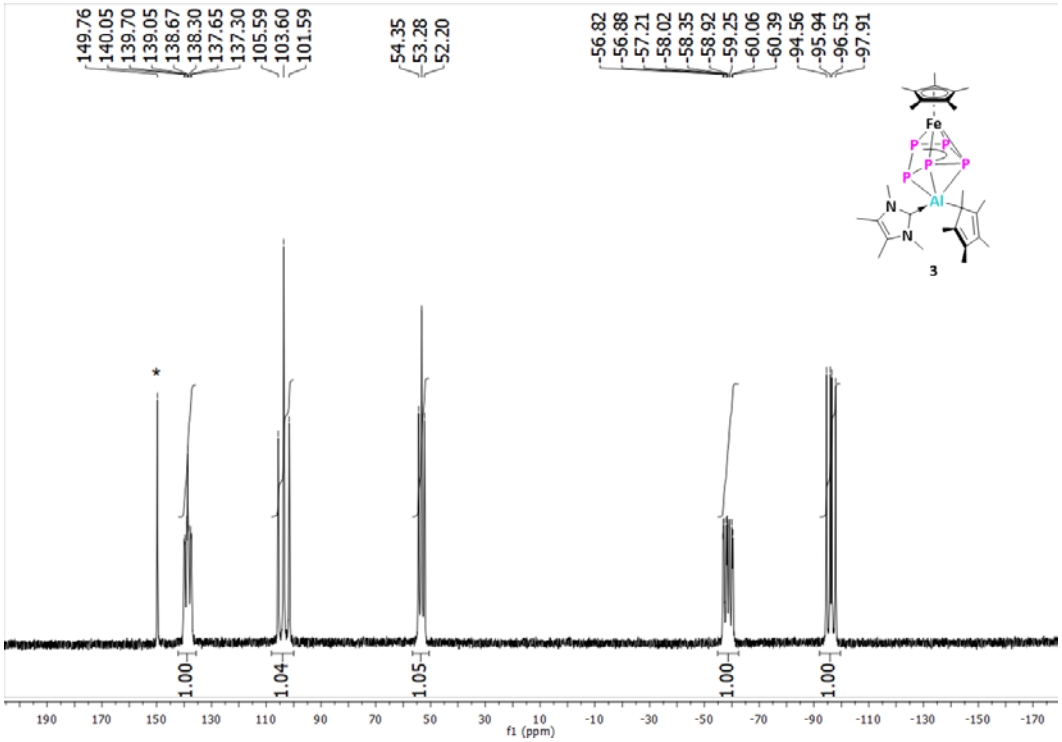

Figure S17. $^{31}P\{^{1}H\}$ NMR (162 MHz, 203 K, toluene-d_8) spectrum of complex **3**. (*****) = traces of [Cp*Fe(η^5-P_5)].

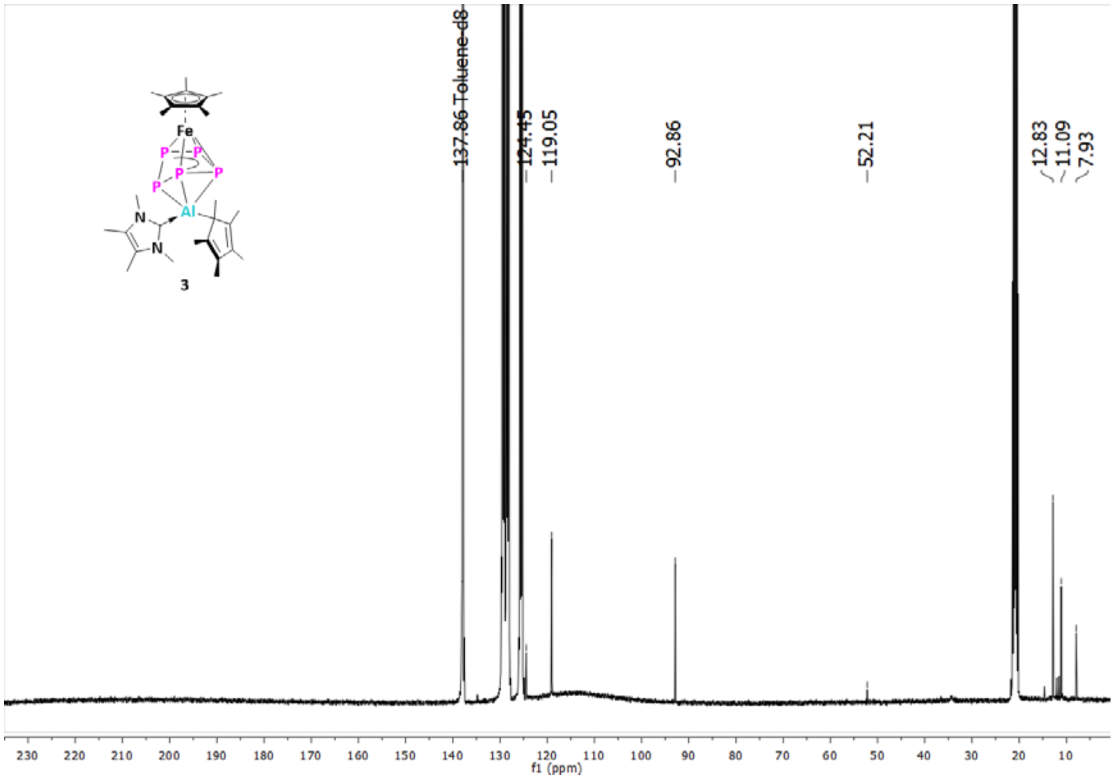

Figure S18. $^{13}C\{^{1}H\}$ NMR (100 MHz, 298 K, toluene-d_8) of complex **3**.

3 Details of the simulations of the $^{31}P\{^1H\}$ NMR spectra.

Simulations of the $^{31}P\{^1H\}$ NMR spectra were performed using the DAISY module of TOPSPIN 3.6 (Bruker). The parameters chemical shift (δ), coupling constant (J) and linewidth ($\omega_{1/2}$) for the simulation of the phosphorus NMR spectra are compiled in the tables provided.

3.1 Experimental and simulated spectra of 1 at 233 K.

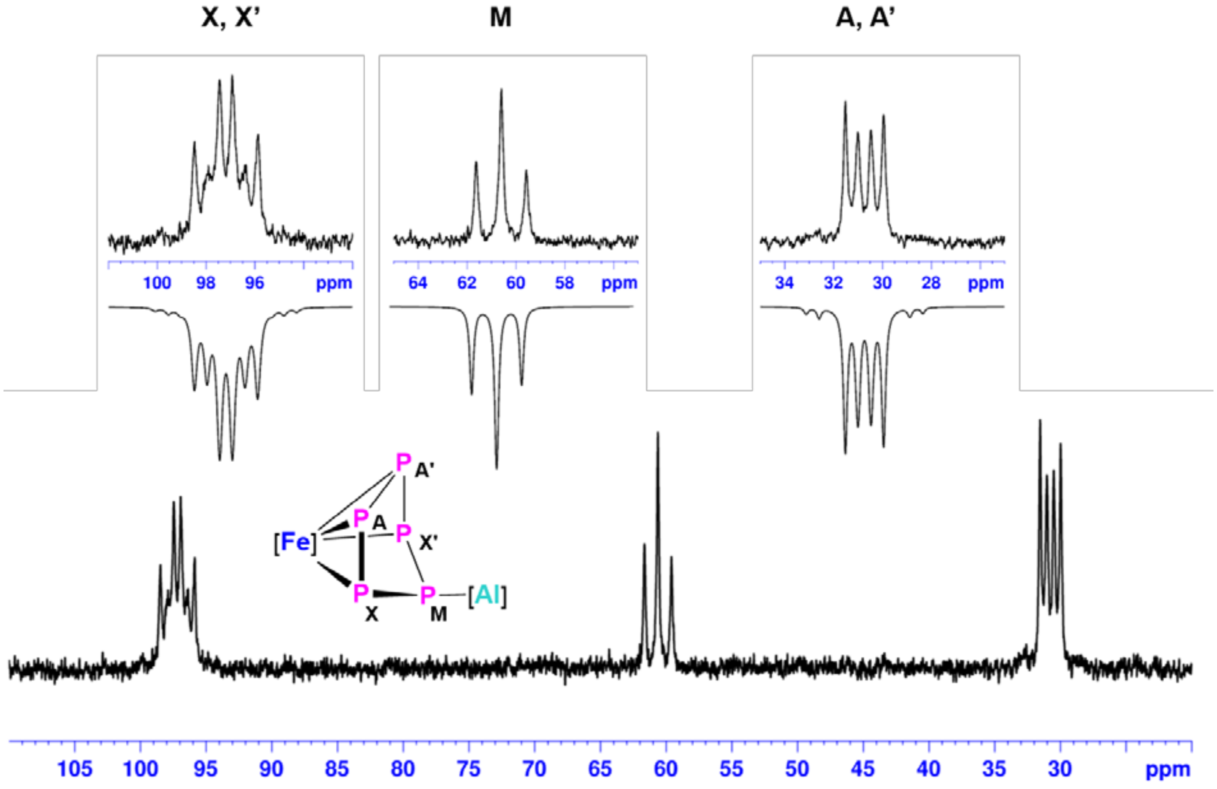

Figure S19. $^{31}P\{^1H\}$ NMR spectrum (161.97 MHz, 233 K, toluene-d_8) of compound **1** with nuclei assigned to an AA'MXX' spin system; insets: extended signals (upwards) and simulations (downwards); $\delta(P_{XX'})$ = 97.2 ppm, $\delta(P_M)$ = 60.6 ppm, $\delta(P_{AA'})$ = 30.7 ppm, $^1J_{AA'}$ = 301.0 Hz, $^1J_{AX}$ = $^1J_{A'X'}$ = 248.2 Hz, $^1J_{MX}$ = $^1J_{MX'}$ = -165.9 Hz, $^2J_{XX'}$ = 48.5 Hz, $^2J_{AX'}$ = $^2J_{A'X}$ = -5.3 Hz, $^2J_{AM}$ = $^2J_{A'M}$ = 3.8 Hz, [Fe] = [Cp*Fe]$^+$, [Al] = [Dipp-*BDI*-Al^{III}]$^{2+}$.

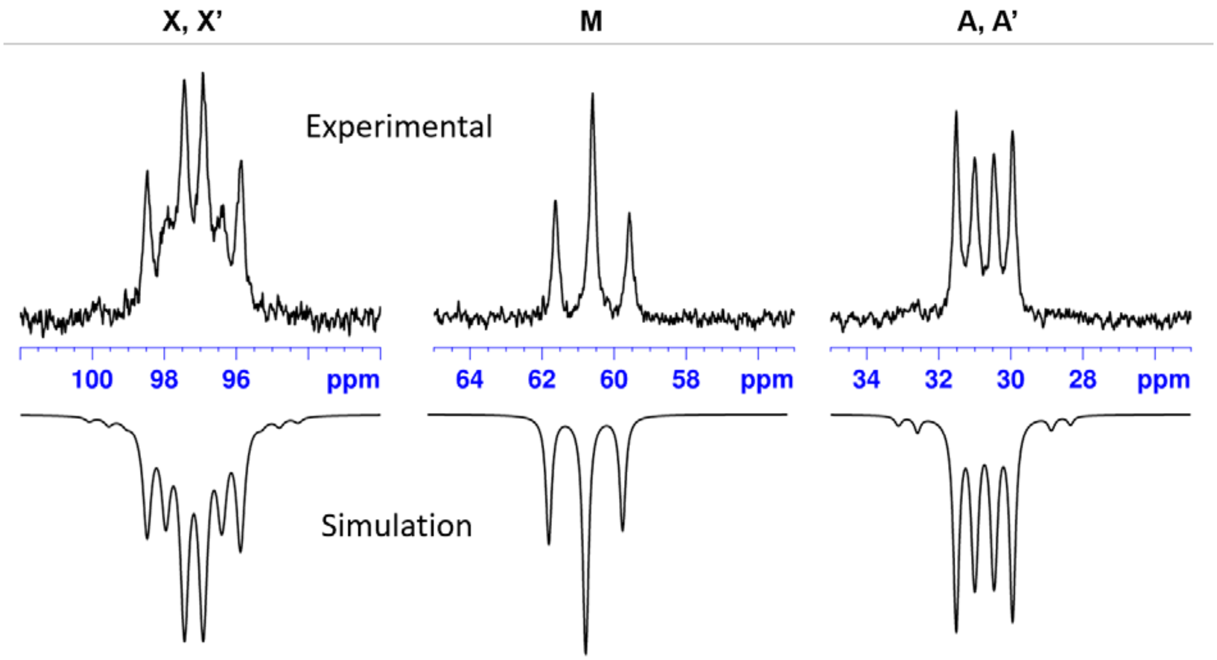

Figure S20. Sections of the $^{31}P\{^{1}H\}$ NMR spectrum (161.97 MHz, 233 K, toluene-d_8) of compound **1** with nuclei assigned to an AA'MXX' spin system; experimental (upwards) and simulation (downwards).

3.2 Table S1. Chemical shifts, coupling constants and linewidths from the iterative fit of the AA'MXX' spin system of **1** at 298 K.

Parameters	Iteration values
$\delta(P_X) = \delta(P_{X'})$	97.17 ppm
$\delta(P_M)$	60.62 ppm
$\delta(P_A) = \delta(P_{A'})$	30.74 ppm
$^1J_{AA'}$	301.0 Hz
$^1J_{AX} = {}^1J_{A'X'}$	248.2 Hz
$^1J_{MX} = {}^1J_{MX'}$	-165.9 Hz
$^2J_{XX'}$	48.5 Hz
$^2J_{AX'} = {}^2J_{A'X}$	-5.3 Hz
$^2J_{AM} = {}^2J_{A'M}$	3.8 Hz
$\omega_{1/2}(X) = \omega_{1/2}(X')$	43.2 Hz
$\omega_{1/2}(M)$	29.6 Hz
$\omega_{1/2}(A) = \omega_{1/2}(A')$	31.3 Hz

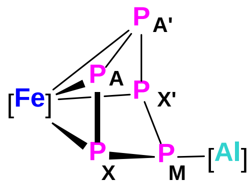

3.3 Experimental and simulated spectra of 2i at 298 K

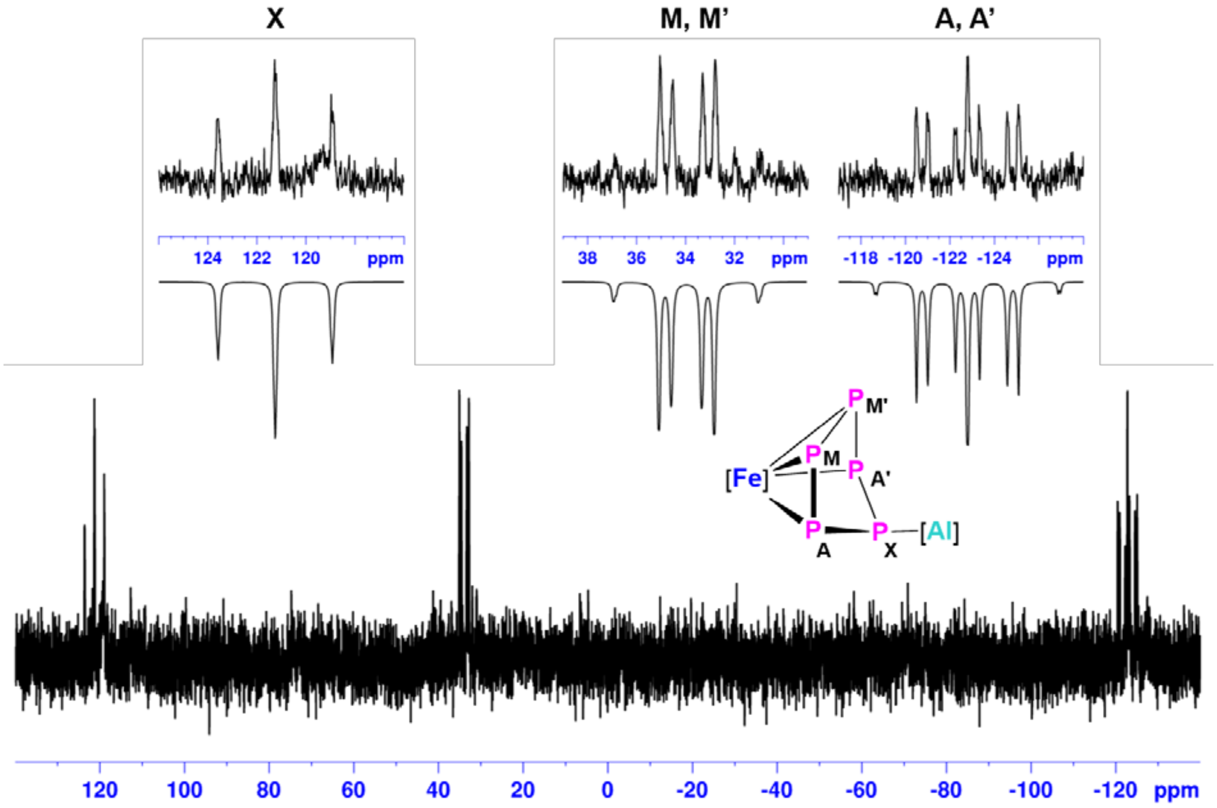

Figure S21. $^{31}P\{^{1}H\}$ NMR spectrum (162 MHz, 298 K, C_6D_6) of compound **2i** with nuclei assigned to an AA'MM'X spin system; insets: extended signals (upwards) and simulations (downwards); $\delta(P_X)$ = 121.3 ppm, $\delta(P_{MM'})$ = 33.9 ppm, $\delta(P_{AA'})$ = -122.8 ppm, $^{1}J_{MM'}$ = 378.2 Hz, $^{1}J_{AM}$ = $^{1}J_{A'M'}$ = 402.8 Hz, $^{1}J_{AX}$ = $^{1}J_{A'X}$ = -377.4Hz, $^{2}J_{AA'}$ = 10.8 Hz, $^{2}J_{A'M}$ = $^{2}J_{AM'}$ = -36.9 Hz, $^{2}J_{MX}$ = $^{2}J_{M'X}$ = -10.2 Hz, [Fe] = Cp*Fe, [Al] = Cp*Al(DME)$_n$.

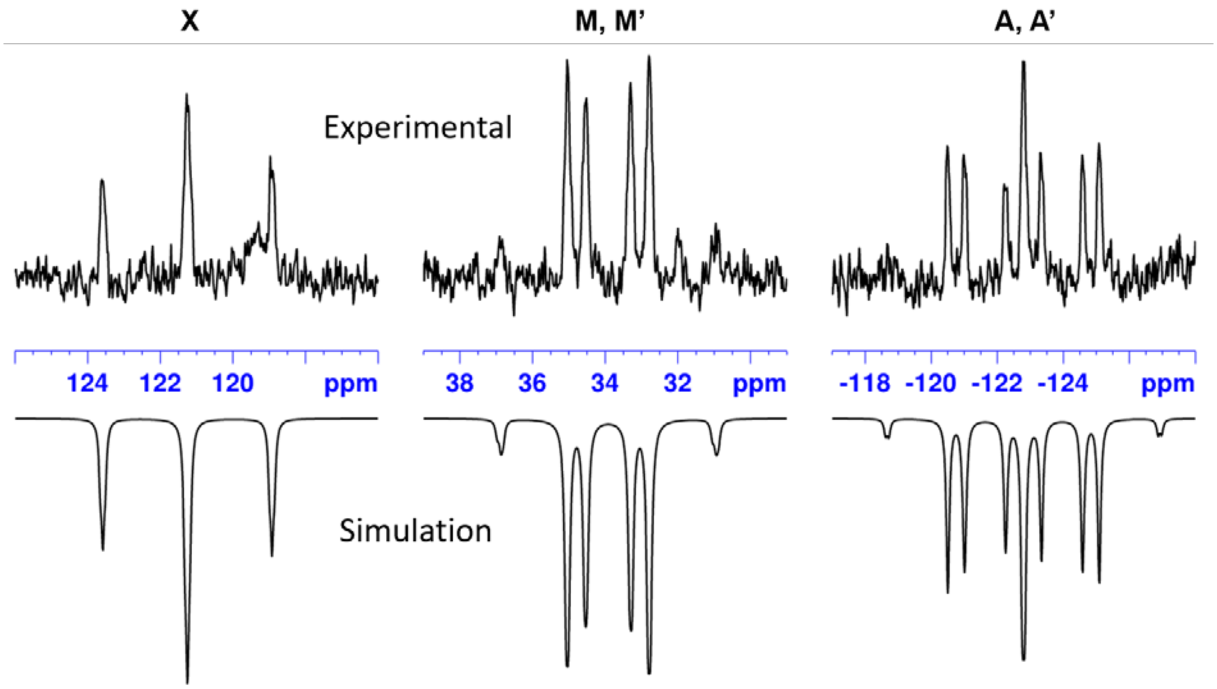

Figure S22. Sections of the $^{31}P\{^{1}H\}$ NMR spectrum (162 MHz, 298 K, C_6D_6) of compound **2i** with nuclei assigned to an AA'MM'X spin system; experimental (upwards) and simulation (downwards).

3.4 Table S2. Chemical shifts, coupling constants and linewidths from the iterative fit of the AMM'XX' spin system of **2i** at 298 K.

N.B. An uncertainty of *ca*. +/- 1 Hz in the refinement data should be noted because of the poor resolution of the spectrum at room temperature.

Parameters	Iteration values
$\delta(P_X)$	121.25 ppm
$\delta(P_M) = \delta(P_{M'})$	33.91 ppm
$\delta(P_A) = \delta(P_{A'})$	-122.78 ppm
$^1J_{MM'}$	378.2 Hz
$^1J_{AM} = {}^1J_{A'M'}$	402.8 Hz
$^1J_{AX} = {}^1J_{A'X}$	-377.4 Hz
$^2J_{AA'}$	10.8 Hz
$^2J_{A'M} = {}^2J_{AM'}$	-36.9 Hz
$^2J_{MX} = {}^2J_{M'X}$	-10.2 Hz
$\omega_{1/2}(X)$	13.8 Hz
$\omega_{1/2}(M) = \omega_{1/2}(M')$	16.2 Hz
$\omega_{1/2}(A) = \omega_{1/2}(A')$	16.1 Hz

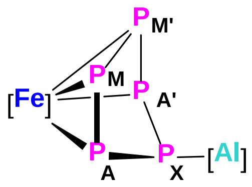

3.5 Experimental and simulated spectra of 3 at 203 K

Two fragments were used for the simulation with statistical weights corresponding to the ratio of the integrals between the two species: complex **3** (weight 1.00), and [Cp*Fe(η^5-P_5)] at δ 149.76 ppm (weight 0.186).

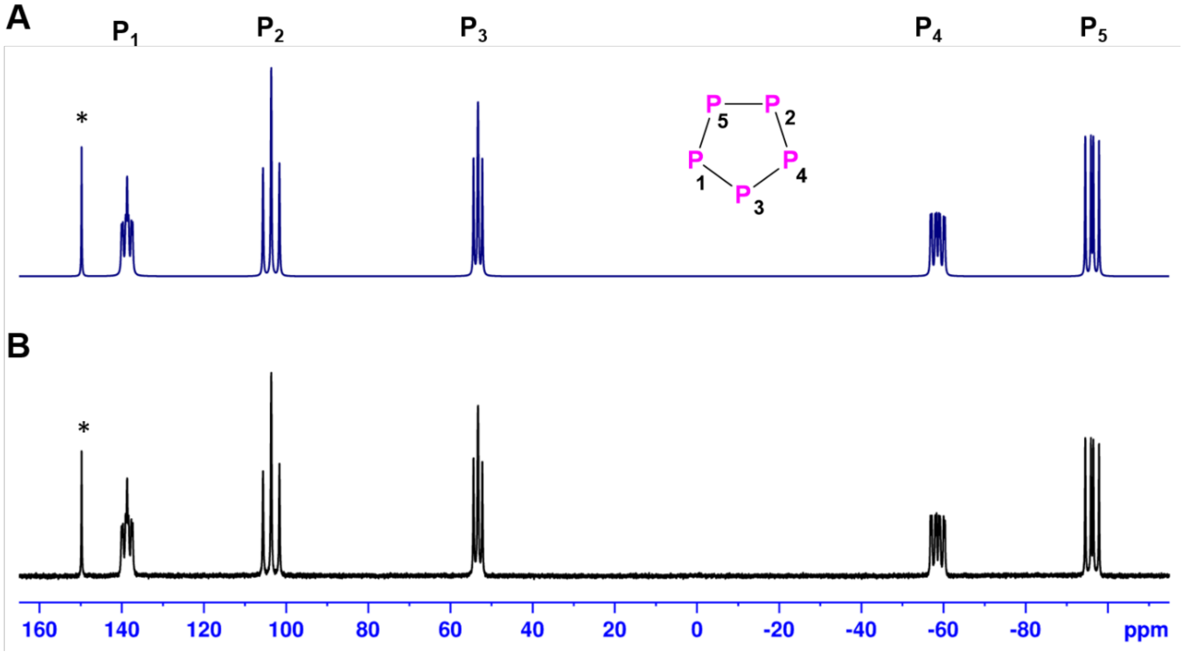

Figure S23. $^{31}P\{^1H\}$ NMR spectrum (162 MHz, 203 K, toluene-d_8) of compound **3** (**B**) and simulation spectrum (**A**). The singlet at δ 149.76 ppm (*) corresponds to trace amounts of [Cp*Fe(η^5-P_5)].

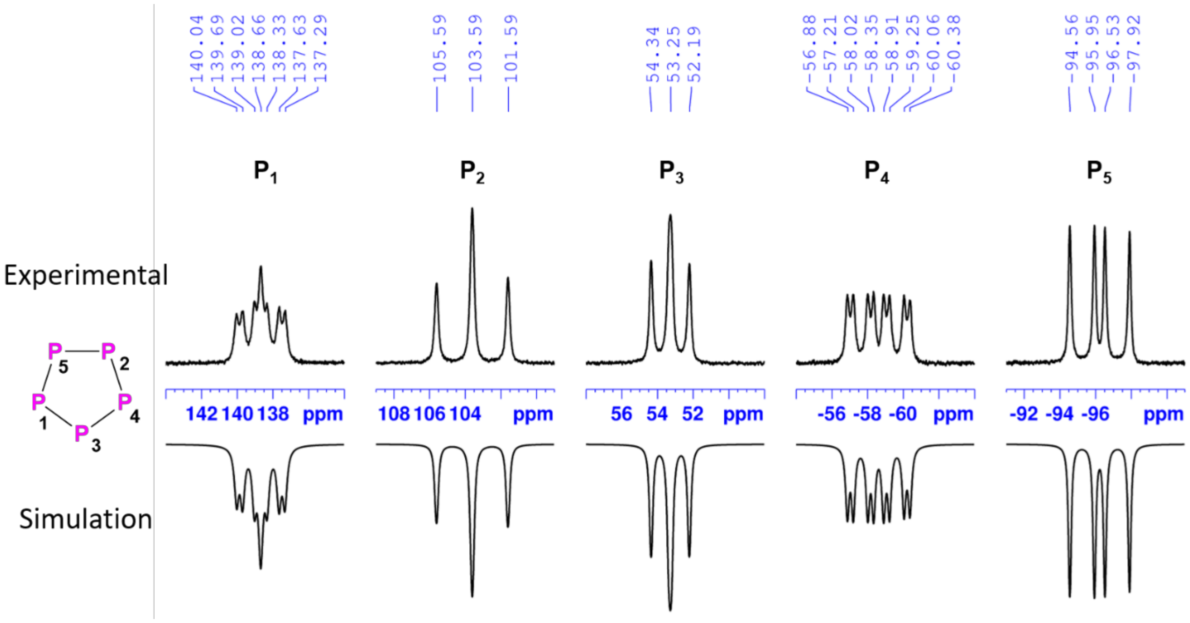

Figure S24. Sections of the $^{31}P\{^{1}H\}$ NMR spectrum (162 MHz, 203 K, toluene-d_8) of compound **3** with assignment of the nuclei; experimental (upwards) and simulation (downwards).

3.6 Table S3. Chemical shifts, coupling constants and linewidths from the iterative fit of the P_5 spin system of complex **3** in toluene-d_8 at 203 K.

Parameters	Iteration values	Parameters	Iteration values
$\delta(P_1)$	138.67 ppm	$^2J_{P1-P2}$	-9.2 Hz
$\delta(P_2)$	103.58 ppm	$^2J_{P1-P4}$	53.7 Hz
$\delta(P_3)$	53.27 ppm	$^2J_{P2-P3}$	-12.0 Hz
$\delta(P_4)$	-58.63 ppm	$^2J_{P3-P5}$	-2.9 Hz
$\delta(P_5)$	-96.23 ppm	$^2J_{P4-P5}$	8.9 Hz
$^1J_{P1-P3}$	161.5 Hz	$\omega_{1/2}(P_1)$	44.2 Hz
$^1J_{P1-P5}$	223.8 Hz	$\omega_{1/2}(P_2)$	30.8 Hz
$^1J_{P2-P4}$	330.0 Hz	$\omega_{1/2}(P_3)$	30.2 Hz
$^1J_{P2-P5}$	319.3 Hz	$\omega_{1/2}(P_4)$	35.7 Hz
$^1J_{P3-P4}$	185.4 Hz	$\omega_{1/2}(P_5)$	26.5 Hz

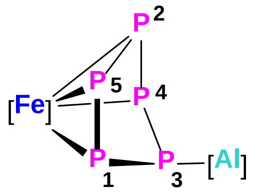

4 IR Spectra

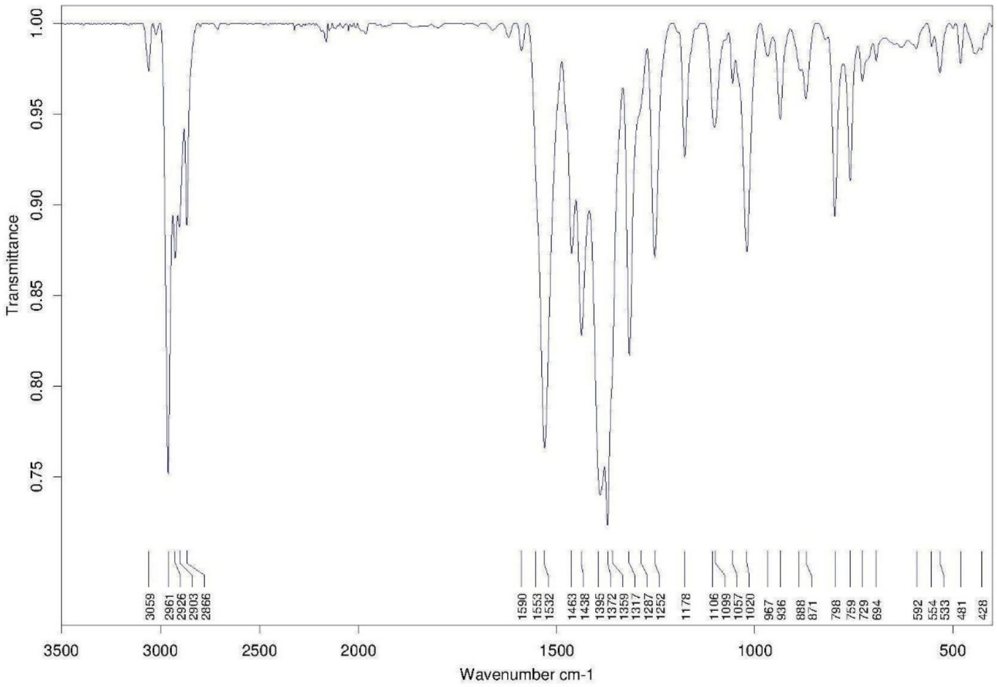

Figure S25. IR spectrum of complex **1**.

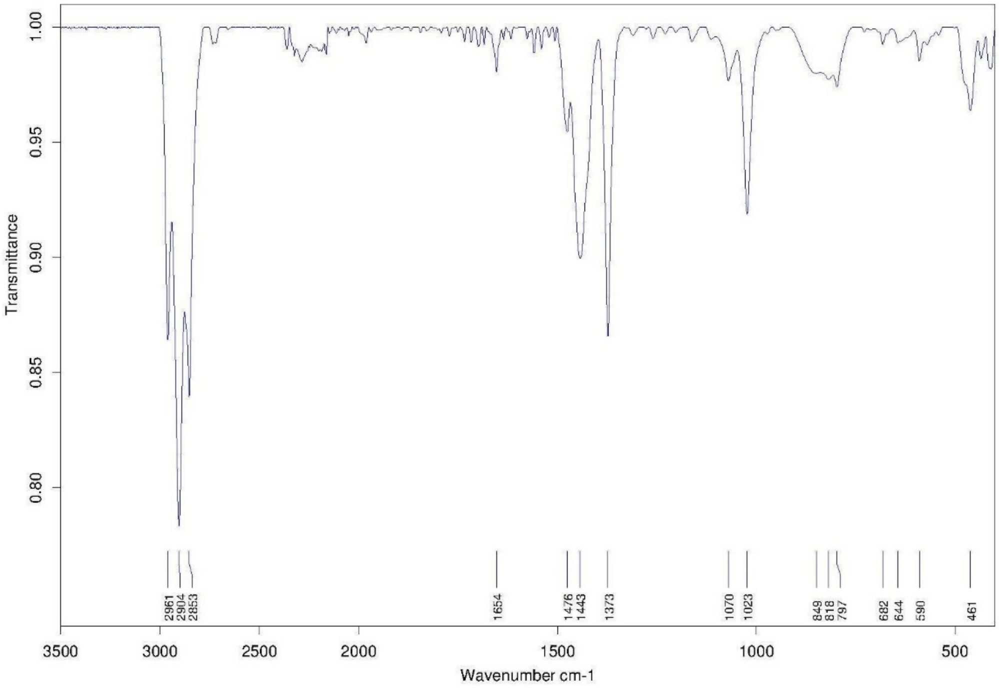

Figure S26. IR spectrum of complex **2**.

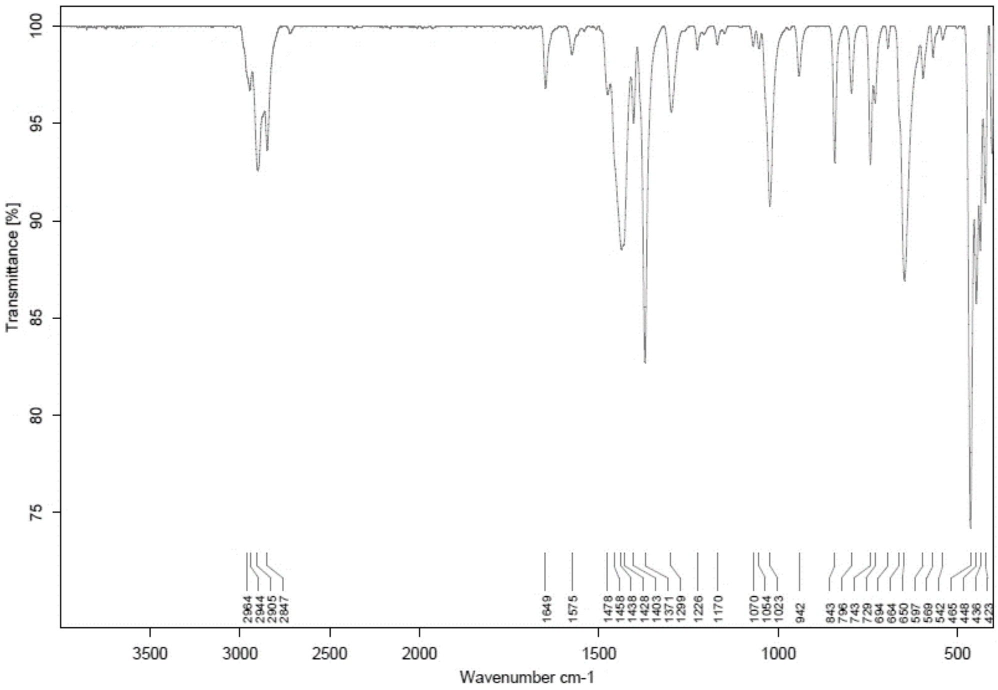

Figure S27. IR spectrum of complex **3**.

5 Single crystal X-ray diffraction

A suitable crystal was covered in mineral oil (Aldrich) and mounted on a glass fiber. The crystal was transferred directly to the cold stream of a STOE IPDS 2 or a STOE StadiVari diffractometer. All structures were solved by using the program SHELXS/T[5] and Olex2.[6] The remaining non-hydrogen atoms were located from successive difference Fourier map calculations. The refinements were carried out by using full-matrix least-squares techniques on F^2 by using the program SHELXL.[5] In each case, the locations of the largest peaks in the final difference Fourier map calculations, as well as the magnitude of the residual electron densities, were of no chemical significance.

Crystallographic data for the structures reported in this paper have been deposited with the Cambridge Crystallographic Data Centre as a supplementary publication no. 1959466 (**1**), 1959467 (**2**), and 1985357 (**3**) Copies of the data can be obtained free of charge on application to CCDC, 12 Union Road, Cambridge CB21EZ, UK (fax: (+(44)1223-336-033; email: deposit@ccdc.cam.ac.uk).

Refinement details

The crystal structure of complex **3** is twined. The twin was solved by using the following twin matrix: TWIN LAW (-1.0, 0.0, 0.0, 0.0, 1.0, 0.0, 0.0, 0.0, -1.0), BASF [0.13(3)]. The Flack value is only 0.046(12) due to twinning, this is leading to one B-Level Alert.

5.1 Table S4. Crystal data and structure refinement

Compound	1	2	3
Formula	$C_{39}H_{56}AlFeN_2P_5$	$C_{40}H_{60}Al_3FeP_5$	$C_{27}H_{42}AlFeN_2P_5$
$D_{calc.}$/ g cm^{-3}	1.295	1.291	1.362
μ/mm^{-1}	0.621	0.628	0.797
Formula Weight	790.53	832.52	632.30
Colour	clear greenish yellow	clear red	clear red
Shape	plate	plate	plate
Size/mm³	0.21×0.13×0.03	0.09×0.06×0.03	0.22×0.12×0.04
T/K	100	100	100
Crystal System	monoclinic	triclinic	orthorhombic
Space Group	$P2_1/n$	*P*-1	*Fdd2*
a/Å	13.1200(9)	11.3626(12)	29.1467(11)
b/Å	23.212(2)	11.7476(12)	50.3825(12)
c/Å	13.6497(11)	16.3303(17)	8.4022(2)
α/°		84.556(9)	
β/°	102.676(6)	88.702(9)	
γ/°		80.771(9)	
V/Å³	4055.6(6)	2141.8(4)	12338.5(6)
Z	4	2	16
Z'	1	1	1
Wavelength/Å	0.71073	0.71073	0.71073
Radiation type	MoK$_\alpha$	MoK$_\alpha$	MoK$_\alpha$
Θ_{min}/°	1.763	1.764	2.425
Θ_{max}/°	30.440	31.824	29.470
Measured Refl.	25656	20959	18451
Independent Refl.	10637	11529	7086
Reflections Used	6295	6147	5099
R_{int}	0.0671	0.0778	0.0475
Parameters	448	462	340
Restraints	0	0	1
Largest Peak	0.615	1.146	1.304
Deepest Hole	-1.010	-0.882	-0.379
GooF	1.023	1.017	0.977
wR_2 (all data)	0.1895	0.2333	0.1309
wR_2	0.1543	0.1858	0.1190
R_1 (all data)	0.1305	0.1653	0.0851
R_1	0.0661	0.0791	0.0538

5.2 Crystal Structures

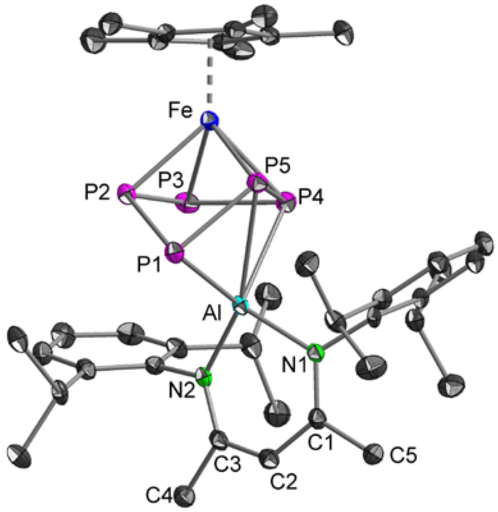

Figure S28. Molecular structure of **1** in the solid state with thermal ellipsoids at the 40% probability. H atoms are omitted for clarity. Selected bond distances (Å) and angles [°]: Fe-P2 2.2578(11), Fe-P3 2.2803(12), Fe-P4 2.3403(12), Fe-P5 2.2110(12), Fe-C30 2.117(4), Fe-C31 2.074(4), Fe-C32 2.088(4), Fe-C33 2.113(4), Fe-C34 2.128(4), P1-P2 2.223(2), P1-P5 2.2145(14), P2-P3 2.1647(14), P3-P4 2.186(2), P4-P5 2.2735(14), P1-Al 2.323(2), P4-Al 2.465(2), P5-Al 2.784(2), Al-N1 1.914(3), Al-N2 1.907(3), N1-C1 1.335(5), N2-C3 1.336(5), C1-C2 1.391(5), C1-C5 1.505(5), C2-C3 1.399(5), C3-C4 1.502(5); P2-P1-Al 90.07(5), P2-P1-P5 82.85(5), P5-P1-Al 75.66(5), P1-P2-P3 107.95(6), P2-P3-Fe 60.99(4), P2-P3-P4 100.67(6), P4-P3-Fe 63.16(4), P3-P4-P5 98.59(5), P3-P4-Al 78.85(5), P5-P4-Al 71.84(5), N2-Al1-N1 96.12(14).

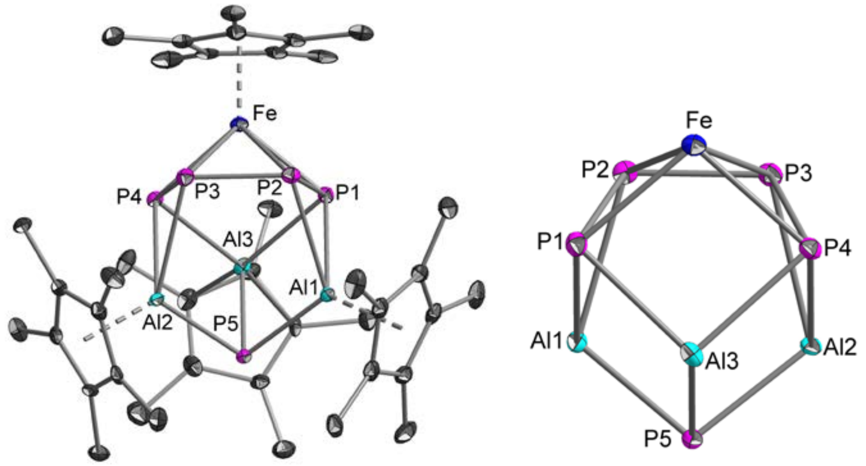

Figure S29. Molecular structure of **2** (left) in the solid state with thermal ellipsoids at the 40% probability and simplified view of the core structure of **2** (right) without the Cp* moieties. H atoms are omitted for clarity. Selected bond distances (Å) and angles [°]: Fe-P1 2.336(2), Fe-P2 2.282(2), Fe-P3 2.289(2), Fe-P4 2.361(2), Fe-C31 2.119(5), Fe-C32 2.116(5), Fe-C33 2.087(5), Fe-C34 2.082(5), Fe-C35 2.088(6), P1-P2 2.193(2), P2-P3 2.192(2), P3-P4 2.189(2), P1-Al1 2.476(2), P1-Al3 2.439(2), P2-Al1 2.676(2), P3-Al2 2.678(2), P4-Al2 2.517(2), P4-Al3 2.417(2), P5-Al1 2.309(2), P5-Al2 2.305(2), P5-Al3 2.334(2), Al1-Al3 2.911(2), Al2-Al3 2.919(2); P1-Fe-P4 97.82(5), P2-Fe-P1 56.68(5), P2-Fe-P3 57.29(5), P2-Fe-P4 99.50(6), P3-Fe-P1 99.97(5), P3-Fe-P4 56.12(5), P1-P2-P3 107.80(8), P2-P3-P4 108.05(7), P1-Al1-P2 50.21(5), P1-Al1-P5 104.59(7), P2-Al1-P5 112.91(7), P3-Al2-P4 49.71(5), P3-Al2-P5 113.34(8), P4-Al2-P5 103.58(7), P1-Al3-P5 105.01(7).

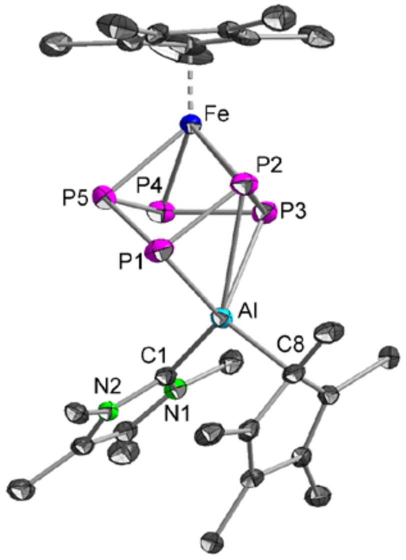

Figure S30. Molecular structure of **3** in the solid state with thermal ellipsoids at the 40% probability. H atoms are omitted for clarity. Selected bond distances (Å) and angles [°]: Fe-P2 2.209(2), Fe-P3 2.348(2), Fe-P4 2.265(2), Fe-P5 2.280(2), Fe-C18 2.129(7), Fe-C19 2.070(7), Fe-C20 2.054(6), Fe-C21 2.105(5), Fe-C23 2.145(6), Al-P1 2.357(2), Al-P2 2.844(2), Al-P3 2.528(2), Al-C1 2.017(6), Al-C8 2.060(6), P1-P2 2.185(3), P2-P3 2.280(3), P3-P4 2.164(3), P4-P5 2.171(3), P5-P1 2.189(3), C1-N1 1.342(7), C1-N2 1.360(7); P2-Fe-P3 59.94(7), P2-Fe-P4 98.01(7), P2-Fe-P5 82.63(7), P4-Fe-P3 55.91(7), P4-Fe-P5 57.06(7), P5-Fe-P3 92.97(7), C1-Al-C8 112.0(2), C1-Al-P1 115.5(2), N1-C1-Al 128.6(4), N2-C1-Al 126.8(4), N1-C1-N2 104.6(5).

6. Quantum Chemical Calculations

Quantum chemical calculations of **1**, **2**, **3** and **2i** were performed using the program package TURBOMOLE.[7] The geometries were determined (without constraint of symmetry for **1**, **2**, **3** and in C_s symmetry for **2i**) using density functional theory (DFT) and the RI-J approximation for the inter-electronic Coulomb term.[8] The functional BP86[9] and basis sets of def2-SV(P) quality were used for all atoms.[10] Shared electron numbers were calculated using the Alhrichs-Heinzmann population analysis[11] based on occupation numbers. They serve as a reliable measure for the covalent bond strength.

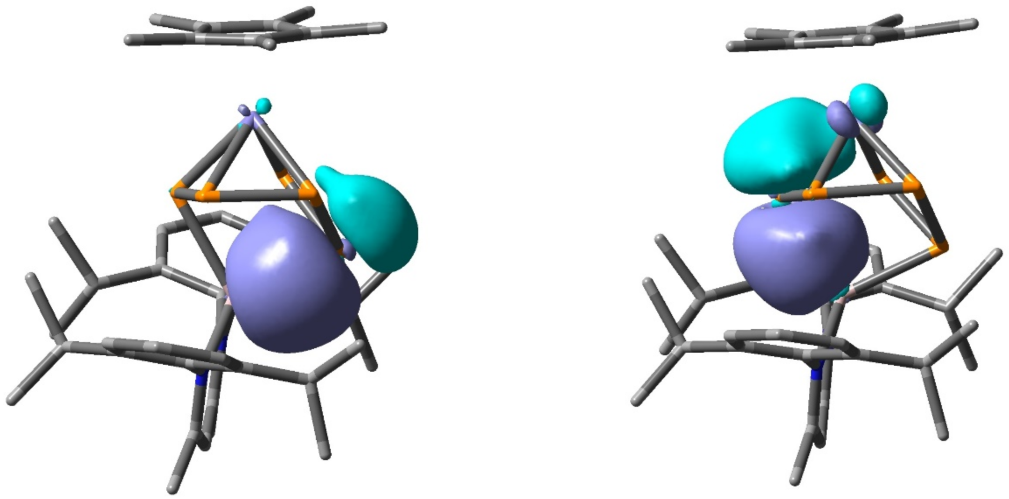

a)

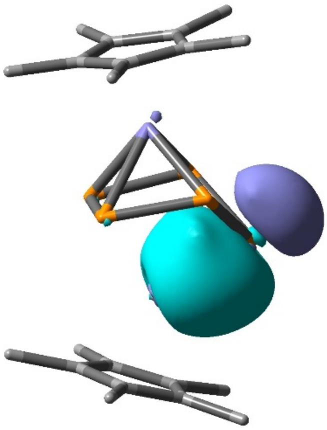

b)

Figure S31: Localized MOs of a) **1** and b) **2i** with pronounced Al-P bonding character (values of isosurfaces ±0.04).

Shared electron numbers of 1, 2, 3 and 2i calculated by means of Ahlrichs-Heinzmann population analyses

1

SEN(P2 - Al) = 0.1322

SEN(P3 - Al) = 0.2551

SEN(P4 - Al) = 1.0379

SEN(P5 - Al) = 0.4532

SEN(P1 - Al) = 1.1085

2i

SEN(Al-P1) = 0.6831

SEN(Al-P2) = 0.2908

SEN(Al-P4) = 0.2908

SEN(Al-P3) = 0.9971

SEN(Al-P5) = 0.9971

3

SEN(Al - P3) = 1.0687

SEN(Al - P2) = 0.3959

SEN(Al - P1) = 0.8958

SEN(Al - P5) = 0.1309

SEN(Al - P4) = 0.2564

2

SEN(P4 - Al3) = 0.9398

SEN(Al3 - P5) = 1.1583

SEN(P1 - Al3) = 0.9544

SEN(P4 - Al2) = 0.7705

SEN(P3 - Al2) = 0.5843

SEN(Al2 - P5) = 1.1772

SEN(P2 - Al1) = 0.5861

SEN(P1 - Al1) = 0.7631

SEN(Al1 – P5) = 1.1786

SEN(Al3 - Al2) = 0.4260

SEN(Al3 - Al1) = 0.4143

SEN(Al2 - Al1) = 0.1481

THREE center SEN:

SEN(P4	P3	Al2)	=	0.2197
SEN(P4	Al3	Al2)	=	0.1403
SEN(P2	P1	Al1)	=	0.2206
SEN(P1	Al3	Al1)	=	0.1426
SEN(Al3	Al2	P5)	=	0.2177
SEN(Al3	Al1	P5)	=	0.2157
SEN(Al2	Al1	P5)	=	0.1412

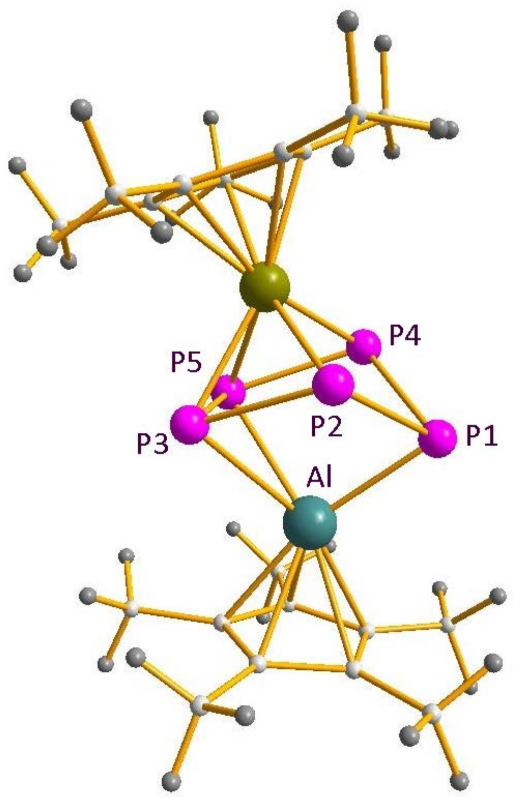

Figure S32: Optimized molecular structure of the hypothetical intermediate **2i**. The atom labels are the same as in the crystal structures of **1** and **3**.

Cartesian coordinates of the molecules under discussion (given in a.u.)

Cartesian coordinates of **1**

-0.33821802340452	-5.95526487209500	0.21937905347384	fe
-4.17936587372500	-4.22813955312787	-0.73046409287115	p
-2.34857475582789	-3.16493369870339	2.88811309142555	p
1.48713885484712	-2.09227533494321	1.55606037905225	p
0.98461679522212	-3.34620117078553	-2.75957004649805	p
1.77805122620575	-9.07264552962863	-1.20550382466675	c
-0.88482147973429	-9.66339642988858	-0.91877910646138	c
-1.52119050745961	-9.37682736028812	1.72922605257771	c
0.73694918993705	-8.53922244362408	3.04463587488144	c
2.77280178987374	-8.39646168293615	1.22816224089699	c
-2.74840468884880	-1.55752763833517	-3.72760152747916	p
-0.71481238473726	1.19152526594064	-0.82272422451950	al
3.28901650183105	-9.30776898880563	-3.59908968234801	c
-2.59622507206282	-10.68310278700672	-2.94325060167775	c
-4.03698082573714	-9.98248914576801	2.89746350178867	c
1.00192982345059	-8.14701984851466	5.84507998770962	c
5.48206100655749	-7.75221153662287	1.78335264575513	c
1.68736546576648	3.53020664728221	-2.42508157649329	n
-2.63796853567808	3.72229429829002	1.07038493104841	n

2.07730643329800	-9.13469697831391	-5.30311782987897	h
4.24876453450801	-11.18315395486521	-3.68803506628529	h
4.77301369151712	-7.82800079173036	-3.74023317936478	h
-2.43314265003525	-12.78301161308924	-3.07402217660890	h
-2.12878272020073	-9.89474516225315	-4.83123376187467	h
-4.60592487191949	-10.22404285564021	-2.55577470971937	h
-4.27584126836581	-9.03127432843283	4.75386214056656	h
-4.25598929027742	-12.05607570233292	3.21076853571300	h
-5.62554479482789	-9.35588025738774	1.67196572294144	h
2.37628722231545	-6.62279008159530	6.28802423631230	h
1.67699949949103	-9.90562578536620	6.79264204160634	h
-0.82325630041948	-7.60520450140184	6.72801234656667	h
6.36960642060196	-6.69761150527395	0.19940810771824	h
6.62303570338949	-9.49939546600942	2.09044328133422	h
5.66278326367328	-6.56823781410654	3.50520875798225	h
1.25546416723382	6.04455938853055	-2.50772792068627	c
3.99285607092833	2.55111879776882	-3.55571371047828	c
-2.46371348006493	6.21996704439976	0.54146971729576	c
-4.33669000515486	2.94527890428965	3.08044310357942	c
-0.77242896773157	7.23919436400545	-1.24726903734629	c
2.99760055554610	7.82501003505701	-3.93733067234527	c
6.23219755424812	2.42799996197271	-2.05922184673054	c
3.99325811775725	1.74433646401613	-6.12893962905007	c

-4.03386176366176	8.13347052253916	1.99148694004081	c
-6.84354696072522	2.16752476440498	2.45149618807151	c
-3.48288209567556	2.98304716512785	5.63552983078130	c
-0.89844459031669	9.29580356110857	-1.52306851629310	h
4.53816697965312	6.83986810103676	-4.94758841326176	h
3.85346004744409	9.21067571101226	-2.60961112800441	h
1.87570146954622	8.93608973478664	-5.32287018041591	h
8.45796046891156	1.50515472829743	-3.19631398713039	c
6.34202074269844	3.32752687664537	0.69012339819792	c
6.28083125120294	0.85568930452450	-7.16415559577671	c
1.64537220704295	1.85523964717642	-7.81898982921182	c
-4.47866253808731	9.78108300548807	0.77242856118004	h
-2.93037607086880	8.85402455365886	3.63087619104153	h
-5.81076015240254	7.33209674497282	2.74908678922539	h
-8.45974155366291	1.44533089929113	4.43993052581855	c
-7.82026713078235	2.14353619735304	-0.27645016468794	c
-5.18671941461364	2.22830701956553	7.54041712768202	c
-0.81633168081724	3.80792912541696	6.40737644202359	c
10.20817637858658	1.40168861200020	-2.07140680951113	h
8.49774242005319	0.73227263133034	-5.72571755860976	c
4.36811715338570	3.63936509620202	1.33623473809112	h
7.76199929769988	5.87151762614079	0.91995192702026	c
7.53656287289219	1.35662834165668	2.47569975353437	c

6.32248504181398	0.23454709279623	-9.15126056191860	h
-0.02808786613065	2.09624982852692	-6.56965297805878	h
1.72133380356747	4.13607856592193	-9.64175738312121	c
1.23813077394297	-0.60150125532129	-9.33767419518436	c
-10.39943487695060	0.83228546110905	4.00332237812998	h
-7.65190464760725	1.47135416986165	6.96157902869869	c
-6.15999806683000	1.81931901798072	-1.52711010283948	h
-9.00595354276719	4.69803248868396	-1.05654550740442	c
-9.71346900431475	-0.00734143590546	-0.80541492098074	c
-4.56029056141054	2.22993882955531	9.52779843927363	h
0.22636225497689	4.33525726038048	4.66072695108495	h
-0.86046912532501	6.14934566480919	8.15385124833066	c
0.67110245617255	1.64843062101115	7.68933778174362	c
10.26287927509239	0.02503218398696	-6.57830649491822	h
9.77328845166375	5.66132442292521	0.33999943632150	h
7.73917321719728	6.55420381725222	2.91003830874189	h
6.91274877233730	7.36452494161728	-0.28563912296012	h
6.53532850504078	-0.48441348767849	2.35079638875862	h
7.41357094926925	2.02051749078462	4.46777416675488	h
9.57256680520134	1.04276363144594	2.04926306170293	h
1.85711988179257	5.96754787094756	-8.62833973817838	h
-0.01790071094101	4.18509006462423	-10.82421493652120	h
3.37420725590912	4.00346701953795	-10.93807029609964	h

2.67815362543190	-0.83387833837986	-10.85445548663995	h
-0.64895852395866	-0.56920596606385	-10.26245291786050	h
1.31693170741856	-2.28886681877508	-8.09074066175379	h
-8.95215627416598	0.88523440551070	8.48075671872232	h
-10.60392955852035	5.20388816565711	0.21654600794217	h
-9.75874667157685	4.57444912160683	-3.01650622240252	h
-7.62501266309507	6.27605396028968	-1.01796423664763	h
-8.96445910550598	-1.86343777163355	-0.16636036126713	h
-10.07183225569603	-0.14032738731275	-2.87214096051697	h
-11.57320791993074	0.32683601706465	0.12178510156417	h
-1.92235392566519	7.75326865500541	7.31075977805553	h
1.09806054064380	6.80567079384228	8.55126680073776	h
-1.76021054389483	5.70630293941776	10.00331947968870	h
-0.23677843805473	1.05449342216675	9.49159631317039	h
2.62782094449362	2.27712930958337	8.13955979622606	h
0.79814529088654	-0.03979109524986	6.44664214297791	h

Cartesian coordinates of **2**

18.01986278037175	13.77778973808880	23.81712608227183	fe
14.52790498330009	11.26806581694090	25.15500661758091	p
14.74677901055492	12.62962449660600	21.17223219189555	p
18.75886913486691	12.05461428554467	19.88056898896446	p
20.87276082727998	10.36893376199698	23.11547704934784	p

17.61526419510309	7.78095554212585	25.18868430065838	al
17.99615510267247	16.22875140609528	27.03351074071427	c
20.55746856173460	15.86848777483062	26.17410970150876	c
20.70623760811142	16.68609888980211	23.57667712378508	c
18.21418846085276	17.53153829364601	22.81095276291325	c
16.54468746872544	17.26560854964011	24.96892907231585	c
13.01760386260709	7.93954984414270	21.96189691456838	al
19.39348623733121	7.04372321497668	19.89633844234911	al
16.20696913480129	4.87174540186799	22.06875735658980	p
18.55296381989784	6.73482494596725	28.86646740662826	c
16.25918683430325	5.18453122466857	29.28983524554270	c
20.54240721808545	4.93141833126418	28.06228785390128	c
17.02910779941046	15.88550987178916	29.68028810249600	c
22.78202867619180	15.06509075859527	27.74301157917522	c
23.08944698421652	16.76809506781557	22.03997170844221	c
17.55597436779657	18.74446618651985	20.33022178793891	c
13.82788442355407	18.06225907762981	25.13662794722958	c
8.96317788774090	7.53852152284795	23.31943026256854	c
9.12791405228566	9.41506820642691	21.33899644020245	c
9.86647217721875	8.13440851236141	19.03234707214608	c
10.03020104030895	5.46928898257633	19.58520330010095	c
9.48653494813222	5.10379246201900	22.20514677193518	c
22.59007676976913	7.51127710604504	17.19654718405065	c

23.18477656077128	5.35843182028016	18.77228954924757	c
21.41637741449403	3.37069373341463	18.17258909442725	c
19.69982685359490	4.30008786705735	16.30593181387488	c
20.37892717124986	6.87618134083953	15.71265987146200	c
19.18208743468937	8.91784945621179	30.63513774339119	c
16.81449618057001	2.69058856634889	28.62150253034950	c
13.87072790967764	6.18608733212263	30.46465645291139	c
19.44579886699752	2.53518049845437	27.86458252686892	c
23.29169877126089	5.61708792765034	27.81718097637533	c
15.05931577247281	15.16064583223149	29.69839947957278	h
17.03741251258817	17.73155704758230	30.69997960996118	h
18.21006272681866	14.55180254476250	30.78318341805297	h
22.19758859650008	14.01115312453198	29.45844174414237	h
23.86877841719144	16.75588801117872	28.38195310144816	h
24.09410131934833	13.84149199543277	26.65418641300809	h
24.14775099932078	14.95534335734916	22.18105779414271	h
24.36255598445366	18.30690044854933	22.71370090544570	h
22.69425208618958	17.10951561817959	20.00909451640180	h
22.69425208618958	17.10951561817959	20.00909451640180	h
18.71195788491325	17.96800102209947	18.75862672273122	h
17.88433602661084	20.82534808352603	20.40534098899852	h
15.54336907735207	18.42753707540187	19.82617550527424	h
13.00227760944919	18.41040299205214	23.24033110580955	h

13.63409115216297	19.83604538372336	26.25796742109570	h
12.65828670931854	16.58353134900472	26.06973777593748	h
8.07782347638896	8.06364378287353	25.97139479016747	c
8.32032688018101	12.12740894429734	21.59212708102709	c
10.11847373100934	9.35848920737532	16.47429390971665	c
10.68538596718592	3.37691423511436	17.78075084059366	c
9.54142928953272	2.58538406464341	23.51958076944031	c
24.19815245631881	9.83835541312158	16.93126731602321	c
25.44559616651146	5.18810127108673	20.49077203158532	c
21.28893780344809	0.77778777921677	19.32612029546082	c
17.56962771983291	2.79466779530937	15.18297221472990	c
19.21703436767539	8.51352377356883	13.69465588606312	c
20.80008867117465	10.05508408347908	29.93197982920700	h
19.70330164353174	8.19060780409068	32.54710110154993	h
17.55340468911214	10.21910337312338	30.87917375059148	h
15.05236943982644	0.46074106731849	28.59520445018246	c
14.11972012520020	6.55694712238831	32.52846454342907	h
12.27782369741109	4.83047637914002	30.27528153863275	h
13.25169332049713	8.00381757627563	29.60050647926013	h
20.67816295895287	0.12496353552928	26.99791109369606	c
24.18094822923756	5.92650840331705	29.70766578355912	h
23.58403543543784	7.38944096106003	26.71606159998099	h
24.39385846445576	4.10682847014675	26.86158712353524	h

8.58147450767759	6.50880391131473	27.28620374298652	h
5.98306788625196	8.29149545059490	26.03529246412626	h
8.93123820974029	9.82678695518816	26.73188033343048	h
8.99022206463327	12.97547902965861	23.39380976510297	h
6.21576628136965	12.27283532895552	21.56365200681770	h
9.05822691595250	13.31404218100461	20.02840803164303	h
10.95156699398169	11.28233532015850	16.61039426970813	h
8.24265491299369	9.54667822843622	15.53211025031454	h
11.34564737206721	8.23636220552241	15.19228770885867	h
11.37809155220240	4.10900823165485	15.94118105836103	h
9.00375496117523	2.17089065240598	17.38111274946266	h
12.18179538696607	2.12656171504541	18.56927231372208	h
8.10252914012358	1.27800243808365	22.70966300601550	h
9.14434391746172	2.76166268614383	25.57131692529280	h
11.42006451100050	1.65439629741761	23.31619462198372	h
23.13412665023936	11.43344368641743	16.08113784449566	h
25.85907711184115	9.44784216896730	15.69040469955919	h
24.92950545188543	10.49411793523918	18.78796684063901	h
25.72683923845245	6.96505018172705	21.57691638718294	h
27.20703813029548	4.83634628167117	19.38825505907646	h
25.25255107874774	3.62875672982848	21.88052547542482	h
22.69798247855636	0.53050066853284	20.85979503774191	h
21.65633217865386	-0.71266053705371	17.88442030756723	h

19.38859615028628	0.40414615084588	20.15355929924678	h
16.50930533690659	1.74093889148744	16.66026163497297	h
18.29061421604284	1.37897953776166	13.79683458709320	h
16.19545573095946	4.01022499222513	14.16489838276156	h
17.21844545684628	7.98938231248151	13.32072239979164	h
20.26157190704479	8.32803961507960	11.87361894598664	h
19.23886210070257	10.54407809315815	14.23209718688508	h
13.11534485526976	0.97227683635449	29.22171249213284	h
15.73016868179720	-1.08196936375801	29.86195383410848	h
14.89098182310859	-0.37350465685413	26.66732517788687	h
22.69025911527740	0.40739399035169	26.46918605183017	h
19.69373772226868	-0.69213804398011	25.32439890055208	h
20.63317850332436	-1.35439427432334	28.49947679987827	h

Cartesian coordinates of **3**

33.21628700697834	56.79478301978587	6.76962042852472	c
35.56627948493421	56.05637035949958	5.99634402200316	n
32.29189079047195	59.47838273355179	9.44418190541249	al
31.59559880435939	55.63244841694494	5.13262222814979	n
37.93712255713491	56.92019043916960	7.10269505544082	c
35.43605013013006	54.47842858788702	3.88486406739043	c
28.78528443437813	62.06975990721092	8.22620855524563	p
31.38559143093945	64.72770760475557	10.23910549102230	p

35.39408806539198	63.09884937898776	9.25514412099380	p
31.98740375580709	57.94209553955279	13.07317752396183	c
34.51219215835017	56.74442193075926	13.11256955502502	c
30.20138167220706	55.93363378434454	12.31452068126419	c
28.84800220173134	55.82954549049021	5.20664317996851	c
32.89842769128127	54.20174728764051	3.33598407990479	c
38.81828399808207	58.38655988342818	5.88390363725460	h
39.24392397279689	55.29861562236429	7.33892232653863	h
37.54351949624771	57.74486467775844	8.98265036202393	h
37.72802791180141	53.41863547804834	2.61913567523645	c
30.74652248206306	63.49644971665428	4.72329989107624	p
33.33059763292089	66.29389989768850	6.85048782271703	fe
34.84627825825060	62.64020929758503	5.12249161494578	p
31.24555047457739	59.93911738864433	15.01672002766987	c
34.24908375695318	54.24542873328174	12.27434499864365	c
36.83253122208473	57.99133077313035	14.19267993218338	c
31.60185944446542	53.74963231853634	11.77766695776020	c
27.37031489110225	56.20887579977936	12.41025068733865	c
27.99350782734777	53.94877029907992	5.57877284746696	h
28.12979014189334	56.58204353047990	3.38476945754907	h
28.30545834666500	57.15573426021550	6.73542628632883	h
31.58898497118878	52.73824658615863	1.30599594877980	c
39.02621358623456	54.93587880815315	1.95704862846525	h

37.17842898654025	52.28405242755384	0.94504301433718	h
38.82961115510225	52.16142219412848	3.89839811519996	h
36.26204445687220	68.67843878269979	5.73444432035105	c
34.14982705485475	68.83797455054003	3.98594827399502	c
31.96121487731146	69.69023482386397	5.39140854674976	c
32.74552934394480	70.12055673403557	7.97775673668817	c
35.38722030828685	69.50827811475696	8.18874225937035	c
32.65076847165964	61.49402638156664	15.13408834686896	h
31.08012875341915	59.07222001560469	16.93401692364136	h
29.39876775156832	60.83109385590772	14.56284211177119	h
36.30085605152242	52.30598422559592	11.92017685257774	c
36.71301347694385	58.13109149669266	16.29534629226898	h
37.09322391749146	59.95508367395898	13.47905159579533	h
38.58614873533242	56.91921985022421	13.75640448925997	h
30.62023375633203	51.24684778689233	10.84384897191055	c
26.71274088644826	58.00689507774079	11.53400663463303	h
26.66989255260977	56.21935603192808	14.40169938960404	h
26.38837786561717	54.63225805618180	11.42839715123105	h
30.28029828866845	51.29138423911618	2.09542965818786	h
32.98612885344873	51.73497875671467	0.10822139413263	h
30.45620445638595	53.98571193958848	0.04527929230727	h
38.94722857846101	68.03176592491950	5.07170021500211	c
34.21979117618145	68.34644063400550	1.19010237763605	c

29.40941589286895	70.29398711544471	4.29948704194093	c
31.12863534841555	71.20134102505068	10.04764864898068	c
36.96308956010839	69.78877810568835	10.53548335157098	c
36.00320820717297	50.62522539432733	13.15754263867579	h
38.20261454650083	53.06962504015835	12.37848151827046	h
36.37209581231936	51.56477381824212	9.94591045992029	h
31.50268173490812	50.66988110141729	9.01595875453943	h
28.53968436613592	51.26945200640532	10.55996793405221	h
31.03588841909650	49.69100335298806	12.20433837758555	h
39.92180971216207	67.05919131243937	6.65729091999236	h
40.05736611357865	69.76439273566885	4.60944328806243	h
39.04508579619666	66.75168233572859	3.41108849283670	h
35.84890075763361	67.13300784844672	0.65975823415653	h
34.38074598115703	70.14385219843020	0.09957124301798	h
32.47824929885964	67.36576223420377	0.53917776092430	h
28.98974348927027	69.10828206984441	2.62029445405028	h
29.30986861020797	72.31415430698122	3.69754740882522	h
27.87073745114393	69.97027199275230	5.68898104326192	h
31.63919815290049	70.42237767762317	11.92952092721106	h
29.09299909048609	70.79680296666517	9.73785965083814	h
31.35324637011957	73.29635721669356	10.14386357038665	h
35.86935427019223	69.31095879612836	12.26344678537648	h
37.64904310414271	71.77104741247184	10.75429390382628	h

38.64510323888259	68.53604101213668	10.49474468333152	h

Cartesian coordinates of **2i**

0.37411699997798	-3.51498550367008	0.00000000000000	fe
1.66402932008094	-7.04270680940216	1.36703088062334	c
1.66402932008094	-7.04270680940216	-1.36703088062334	c
-0.88601295144160	-6.58183253695632	-2.21615278226242	c
-2.46472136319722	-6.24515277868702	0.00000000000000	c
-0.88601295144160	-6.58183253695632	2.21615278226242	c
-1.70429148444496	-0.26812549530629	-2.12274704276178	p
2.46235805536777	-1.12331916601118	-2.84300400222419	p
4.64393159317876	1.18858738328290	0.00000000000000	p
2.46235805536777	-1.12331916601118	2.84300400222419	p
-1.70429148444496	-0.26812549530629	2.12274704276178	p
3.88376883359103	-7.65872441047455	3.02794016514238	c
5.69829434061903	-7.06060865797058	2.16035997042396	h
3.99674393593564	-9.73748786217414	3.36701709900149	h
3.74202030295638	-6.71819981768372	4.89749347956886	h
-1.73654000010390	-6.57197680944879	4.92566272980607	c
-5.28228248437771	-5.88678165671647	0.00000000000000	c
-1.73654000010390	-6.57197680944879	-4.92566272980607	c
3.88376883359103	-7.65872441047455	-3.02794016514238	c
-0.34695510157959	-5.60271719828065	6.16936290277693	h

-5.92654100587925	-4.81646824753511	1.68777155405703	h
-3.57473763284360	-5.58733430677965	-5.15755934575240	h
3.74202030295638	-6.71819981768372	-4.89749347956886	h
5.69829434061903	-7.06060865797058	-2.16035997042396	h
-0.34695510157959	-5.60271719828065	-6.16936290277693	h
-5.92654100587925	-4.81646824753511	-1.68777155405703	h
-3.57473763284360	-5.58733430677965	5.15755934575240	h
-1.96892552104190	-8.53630869703740	5.65467569007502	h
-6.28508665292883	-7.74057649360744	0.00000000000000	h
-1.96892552104190	-8.53630869703740	-5.65467569007502	h
3.99674393593564	-9.73748786217414	-3.36701709900149	h
0.74505720667391	3.37580979038533	0.00000000000000	al
1.73707985406503	7.33299024465405	-1.35933049025511	c
-0.77492737021654	6.67386029346508	-2.20925687971054	c
-2.33492868182322	6.29707372903406	0.00000000000000	c
-0.77492737021654	6.67386029346508	2.20925687971054	c
1.73707985406503	7.33299024465405	1.35933049025511	c
4.00083726235076	7.92383666612002	-2.97559329368431	c
-1.67573969604460	6.55845937586513	-4.90726997066964	c
-5.11734967773475	5.71270091362343	0.00000000000000	c
-1.67573969604460	6.55845937586513	4.90726997066964	c
4.00083726235076	7.92383666612002	2.97559329368431	c
5.68936615526110	6.82128401854994	-2.37383996575294	h

-0.09921008243575	6.19895549504294	-6.24464773041885	h
-5.68078973743345	4.60105398402856	-1.68972948825153	h
-3.08990159572369	5.03227694238914	5.19317268228026	h
3.65419938376031	7.49132106724515	4.99855525613397	h
5.68936615526110	6.82128401854994	2.37383996575294	h
-0.09921008243575	6.19895549504294	6.24464773041885	h
-5.68078973743345	4.60105398402856	1.68972948825153	h
-3.08990159572369	5.03227694238914	-5.19317268228026	h
3.65419938376031	7.49132106724515	-4.99855525613397	h
4.50972605532323	9.96395379070694	-2.84760420656806	h
-2.58168343423904	8.37343877922464	-5.47695907541249	h
-6.26307271153530	7.48208333189307	0.00000000000000	h
-2.58168343423904	8.37343877922464	5.47695907541249	h
4.50972605532323	9.96395379070694	2.84760420656806	h

7 References

[1] C. Cui, H. W. Roesky, H.-G. Schmidt, M. Noltemeyer, H. Hao, F. Cimpoesu, *Angew. Chem. Int. Ed.* **2000**, *39*, 4274.

[2] S. Schulz, H. W. Roesky, H. J. Koch, G. M. Sheldrick, D. Stalke, A. Kuhn, *Angew. Chem. Int. Ed.* **1993**, *32*, 1729.

[3] O. J. Scherer, T. Brück, *Angew. Chem. Int. Ed.* **1987**, *26*, 59.

[4] N. Kuhn, T. Kratz, *Synthesis* **1993**, *1993*, 561.

[5] a) G. Sheldrick, *Acta Crystallogr., Sect. A* **2008**, *64*, 112; b) G. Sheldrick, *Acta Crystallogr., Sect. C* **2015**, *71*, 3.

[6] O. V. Dolomanov, L. J. Bourhis, R. J. Gildea, J. A. K. Howard, H. Puschmann, *J. Appl. Cryst.* **2009**, *42*, 339.

[7] TURBOMOLE 7.0, TURBOMOLE GmbH 2015. University of Karlsruhe and Forschungszentrum Karlsruhe, 1989-2007, TURBOMOLE GmbH since 2007.

[8] M. Sierka, A. Hogekamp, R. Ahlrichs, *J. Chem. Phys.* **2003**, *118*, 9136.

[9] a) A. D. Becke, *Phys. Rev. A* **1988**, *38*, 3098; b) J. P. Perdew, *Phys. Rev. B* **1986**, *33*, 8822.

[10] a) F. Weigend, R. Ahlrichs, *Phys. Chem. Chem. Phys.* **2005**, *7*, 3297; b) F. Weigend, *Phys. Chem. Chem. Phys.* **2006**, *8*, 1057.

[11] a) R. Heinzmann, R. Ahlrichs, *Theor. Chim. Acta* **1976**, *42*, 33; b) R. Ahlrichs, C. Ehrhardt, *Chem. Unserer Zeit* **1985**, *19*, 120.
